# Supplementary material for: S100PBP interacts with nucleoporin TPR and facilitates XY crossover formation in mice
Source: EMBO Rep. 2025 Apr 9;26(9):2280–99. doi: 10.1038/s44319-025-00391-y (PMC12069632; doi:10.1038/s44319-025-00391-y)
Supplement: Supplementary file 1 — Appendix [file 44319_2025_391_MOESM1_ESM.pdf]

Appendix for

S100PBP interacts with nucleoporin TPR and facilitates XY crossover formation in mice

Table of content

Title page	Page 1
Appendix Figure S1&S2	Page 2
Appendix Figure S3&S4	Page 3
Appendix Table S1&S2	Page 4

Appendix Figure S1

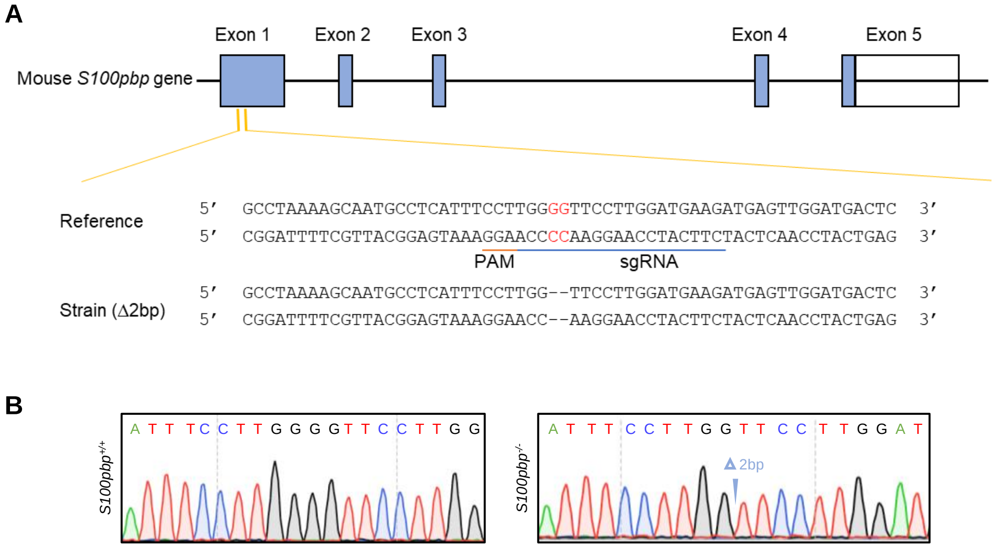

Appendix Figure S1. Generation of *S100pbp*$^{-/-}$ mice.

(**A**) The strategy to generate *S100pbp*$^{-/-}$ mice. The single guide RNA (sgRNA, underlined) was designed to target exon 1 of the *S100pbp* gene. A 2-base-pair deletion was detected in the *S100pbp* knockout allele. PAM, protospacer adjacent motif. (**B**) Sanger sequencing confirmed the 2-base-pair deletion in *S100pbp*$^{-/-}$ mice at the genomic DNA level. The arrowhead indicates the mutation site.

Appendix Figure S2

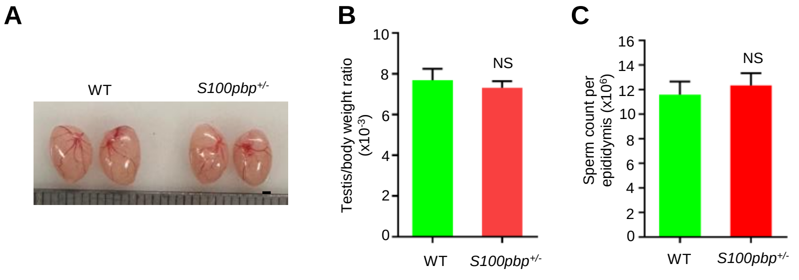

Appendix Figure S2. *S100pbp*$^{+/-}$ mice show normal spermatogenesis.

(**A**) Representative images of testes from 8-week-old control and *S100pbp*$^{+/-}$ mice. Each grid represents 1 mm. (**B** and **C**) The ratio of testis/body weight (**B**) and sperm count per epididymis (**C**) of 8-week-old control and *S100pbp*$^{+/-}$ mice. Data represent the mean ± SEM from at least three mice analyzed for each genotype. Statistical significance was determined using Student's *t* -test. NS, not significant.

Appendix Figure S3

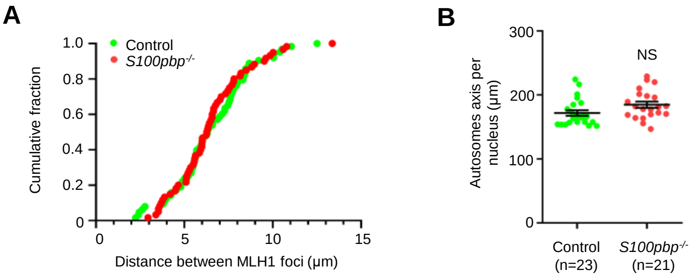

Appendix Figure S3. Crossover interference and axis length are unchanged in *S100pbp*$^{-/-}$ spermatocytes.

(A) Analyses of crossover interference in *S100pbp* mutant versus control spermatocytes (mid-pachytene). Distances between MLH1 foci were measured on autosomal bivalents containing 2 or 3 foci. n, the number of bivalents analyzed from at least biological replicates. (B) A scatter plot showing the length of autosomal axes per nucleus. Data represent the mean ± SEM from at least three biological replicates. n, the number of mid-pachytene nuclei analyzed. Statistical significance was determined using Student's *t*-test. NS, not significant.

Appendix Figure S4

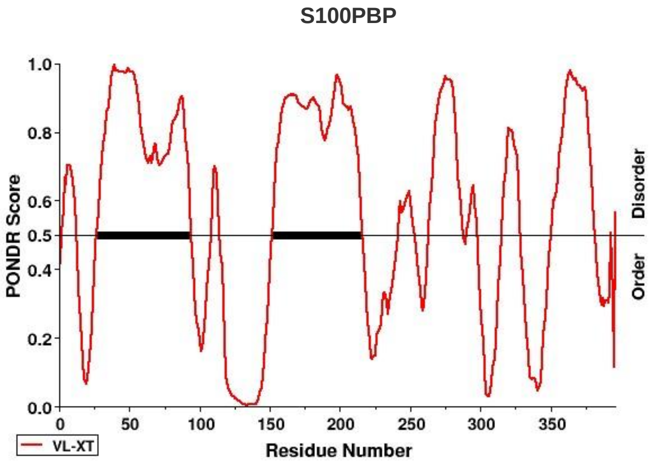

Appendix Figure S4. Prediction of intrinsic disorder regions.

Graphs plotting intrinsic disorder regions (PONDR VL-XT) of S100PBP. PONDR VL-XT score (y-axis) and amino acid position (x-axis) are shown.

Appendix Table S1. Male mouse fertility assay

Genotype	Mating period (months)	No. of fertile mice/No. of mice tested	The average litter size /male/month	The average number of pups/litter
Control	3	3/3	1.78 ± 0.15	7.74 ± 0.35
S100pbp$^{-/-}$	3	0/3	0	0

Each male mouse (8-10 weeks of age) was caged with two wild-type female mice (8-10 weeks of age) for three months.

Appendix Table S2. Antibodies used in this study

Protein	Host species	Company	Catalog number	Dilution
S100PBP	Rabbit	Made by Abclonal	-	WB:1:1000; IF: 1:100; IP: 1:200
	Rat	Made by Abclonal	-	WB:1:500
GFP	Mouse	Abmart	M20004	WB: 1:3000
FLAG	Rabbit	Proteintech	4K14	IF: 1:100
	Mouse	Abmart	M20008	WB: 1:3000
GAPDH	Mouse	Proteintech	60004-1-Ig	WB: 1:3000
β-Actin	Rabbit	Abcam	ab8227	WB 1:3000
RAD51	Rabbit	Made by Abclonal	-	IF: 1:50
RPA2	Rat	Cell Signaling Technology	2208	IF: 1:100
MLH1	Rabbit	Made by Abclonal	-	IF: 1:50
MLH3	Rabbit	gifted from Dr. Mengcheng Luo	-	IF: 1:50
MSH4	Rabbit	Made by Duoneng	-	IF: 1:50
TPR	Mouse	Santa Cruz Biotechnology	3B9	IF:1:100
	Rabbit	NOVUS	NB100-2866	WB 1:1000
γH2AX	Rabbit	NOVUS	NB100-384	IF: 1:5000
M1AP	Rabbit	Made by Abclonal	-	IF: 1:50
TEX11	Rabbit	Made by Abclonal	-	IF: 1:50
H1t	Guinea pig	Made by Dia-An	-	IF: 1:200
SYCP2	Guinea pig	Made by Abclonal	-	IF: 1:500
CREST	Human	Immunovision	HCT-0100	IF: 1:500
SIX6OS1	Rat	Made by Abclonal	-	IF: 1:200
SYCP3	Mouse	Abcam	ab97672	IF: 1:100

WB, western blotting. IF, immunofluorescence staining. IP, immunoprecipitation.
