# Supplementary figures and images for: S100PBP interacts with nucleoporin TPR and facilitates XY crossover formation in mice
Source: EMBO Rep. 2025 Apr 9;26(9):2280–99. doi: 10.1038/s44319-025-00391-y (PMC12069632; doi:10.1038/s44319-025-00391-y)

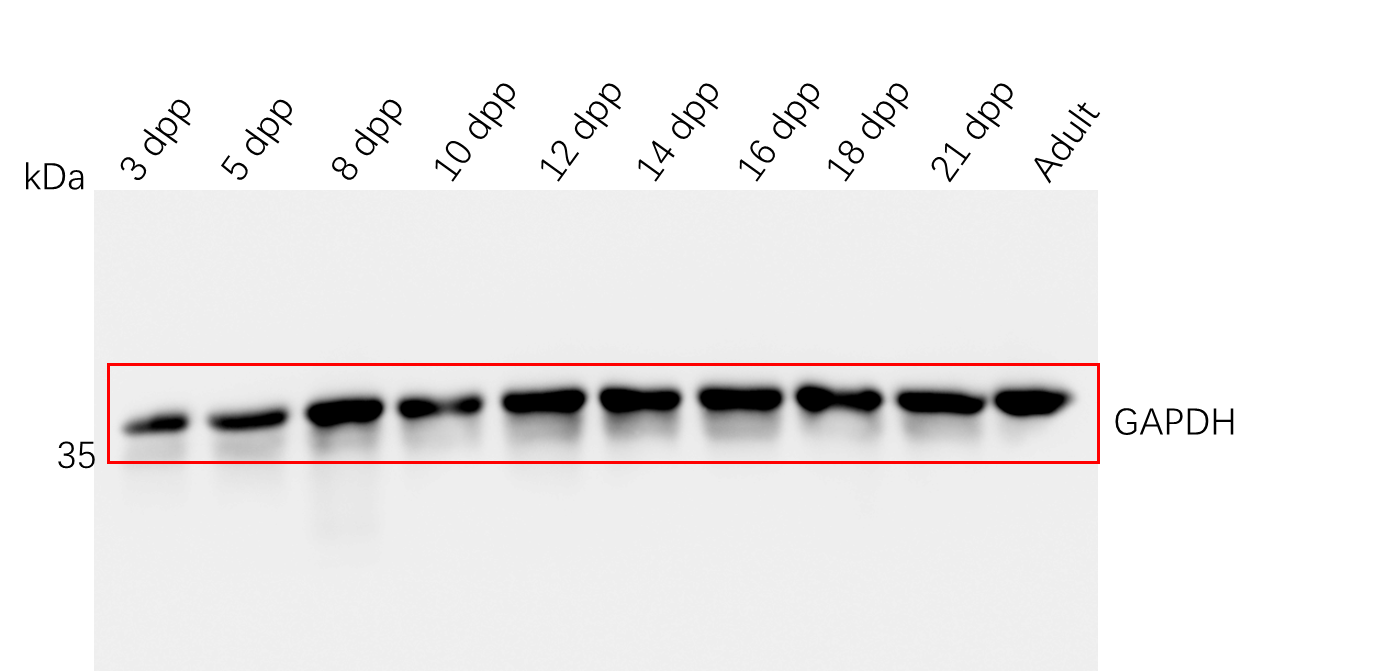

Supplement: Supplementary file 4 — Source data Fig. 1 [file 44319_2025_391_MOESM4_ESM.zip › Fig.1/B/GAPDH.png]

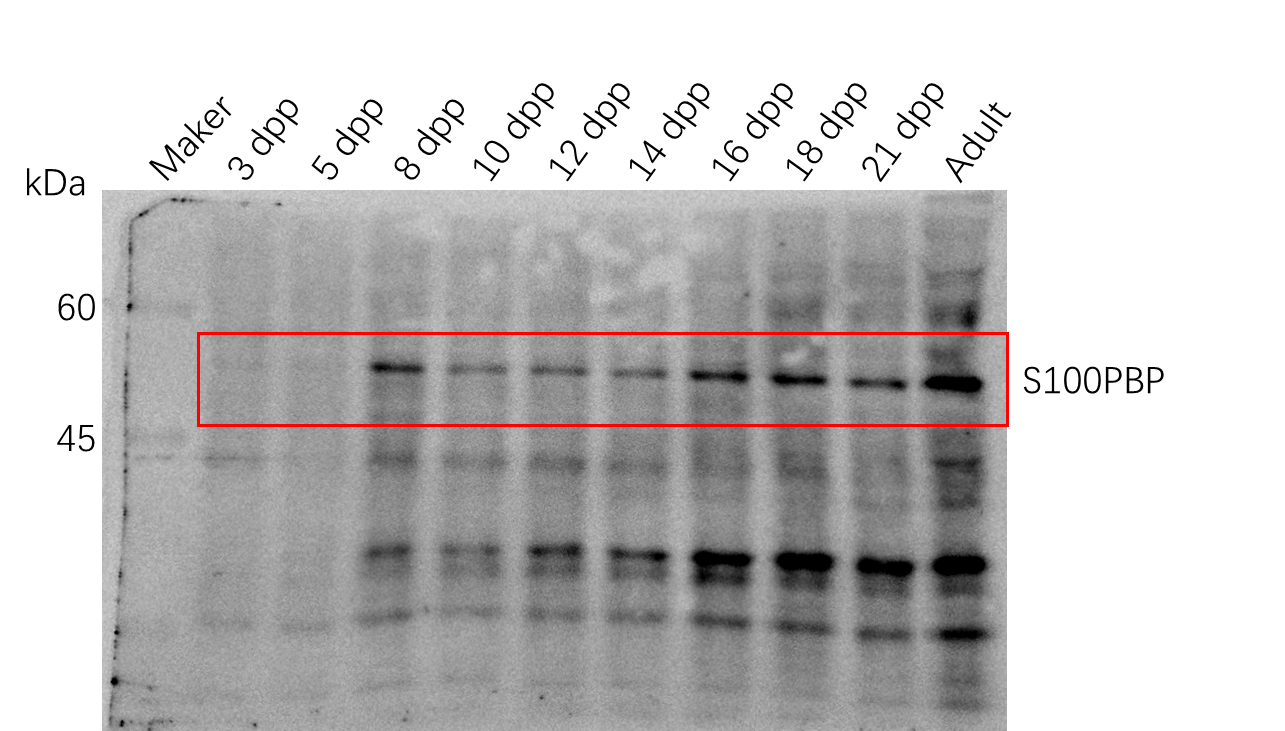

Supplement: Supplementary file 4 — Source data Fig. 1 [file 44319_2025_391_MOESM4_ESM.zip › Fig.1/B/S100PBP.png]

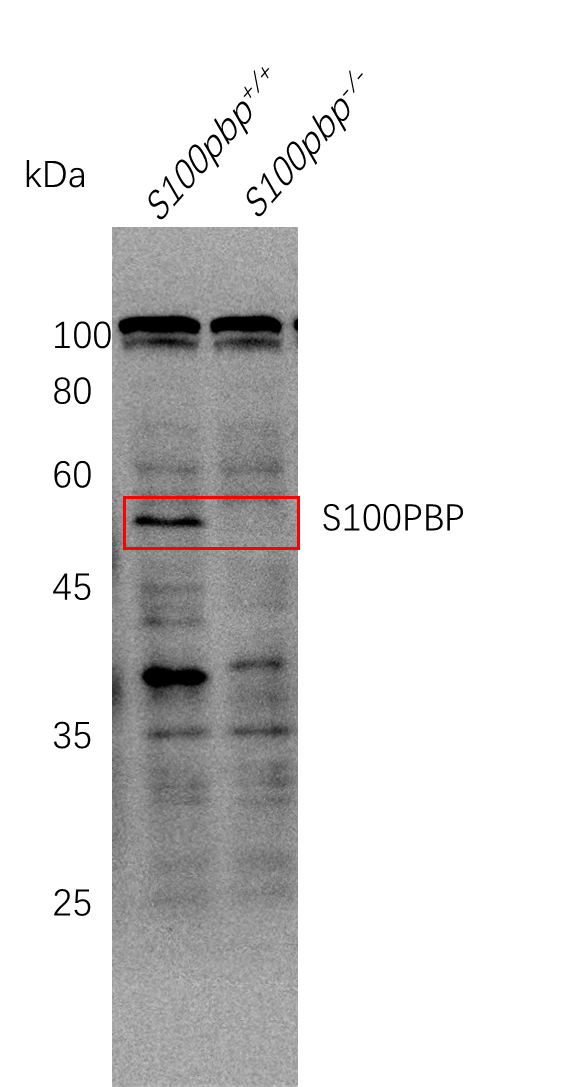

Supplement: Supplementary file 4 — Source data Fig. 1 [file 44319_2025_391_MOESM4_ESM.zip › Fig.1/C/S100PBP.png]

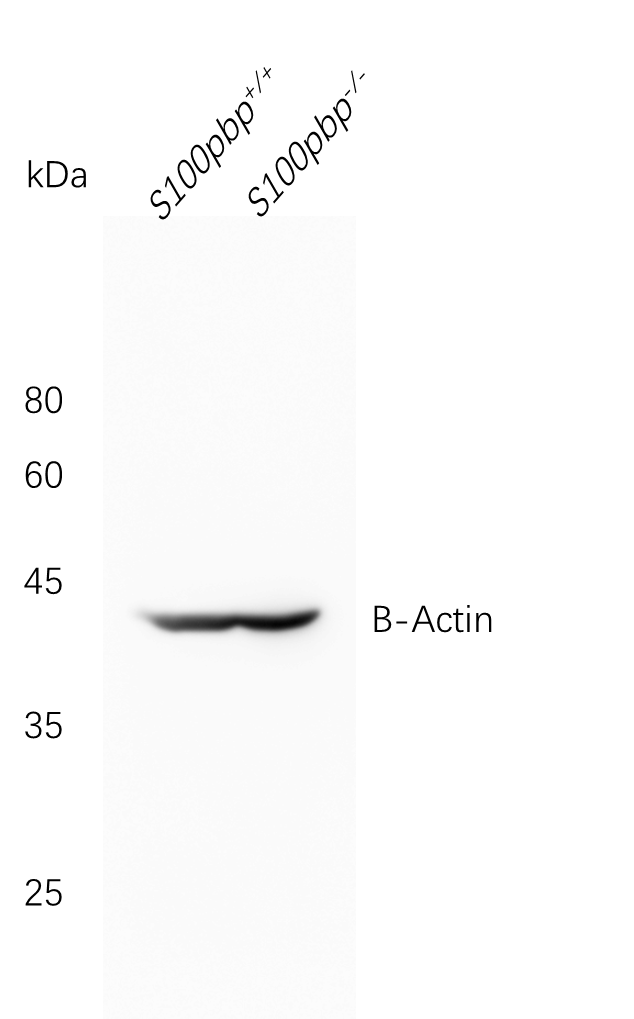

Supplement: Supplementary file 4 — Source data Fig. 1 [file 44319_2025_391_MOESM4_ESM.zip › Fig.1/C/β-Actin.png]

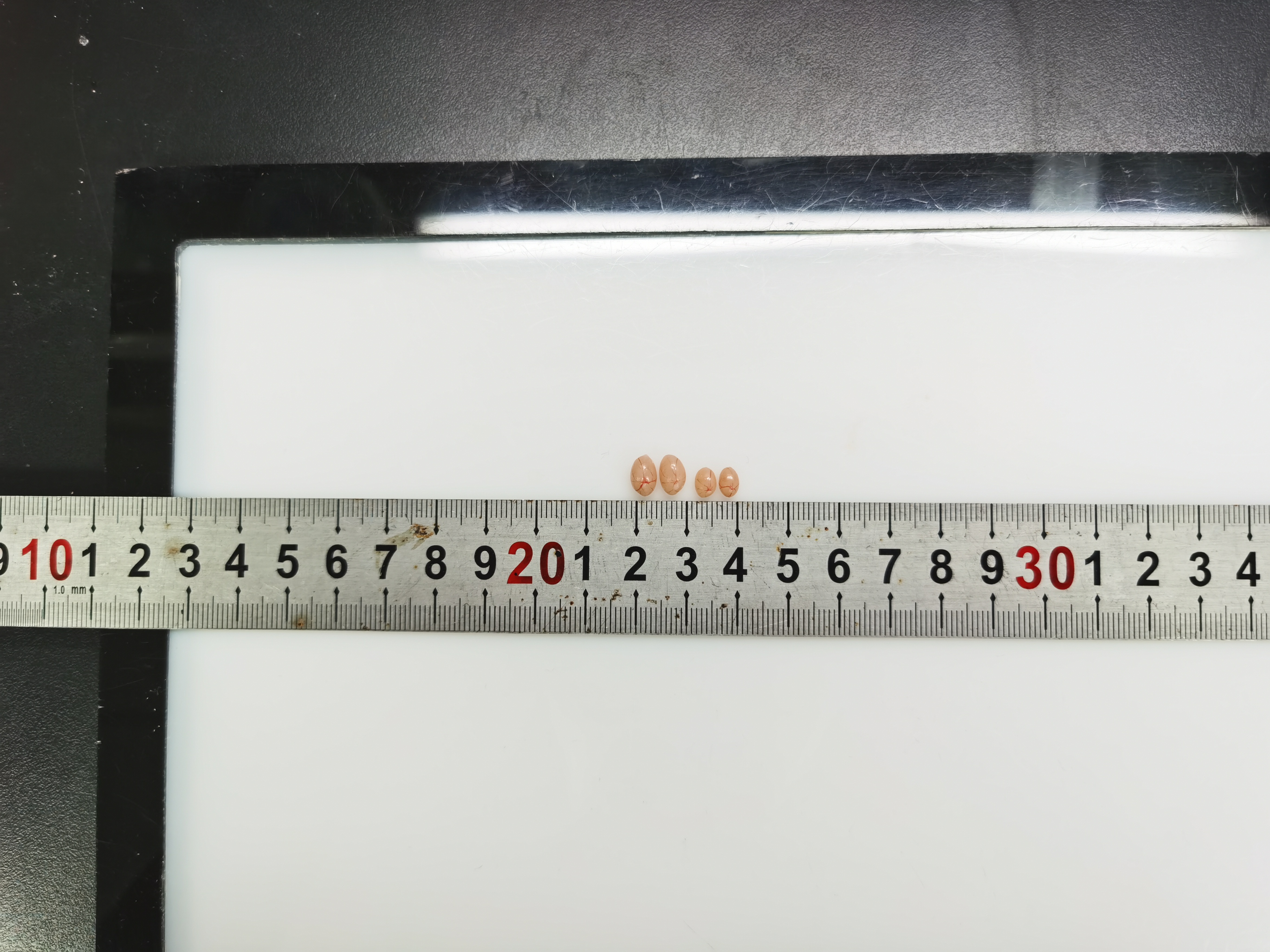

Supplement: Supplementary file 4 — Source data Fig. 1 [file 44319_2025_391_MOESM4_ESM.zip › Fig.1/D/左Control,右KO.jpg]

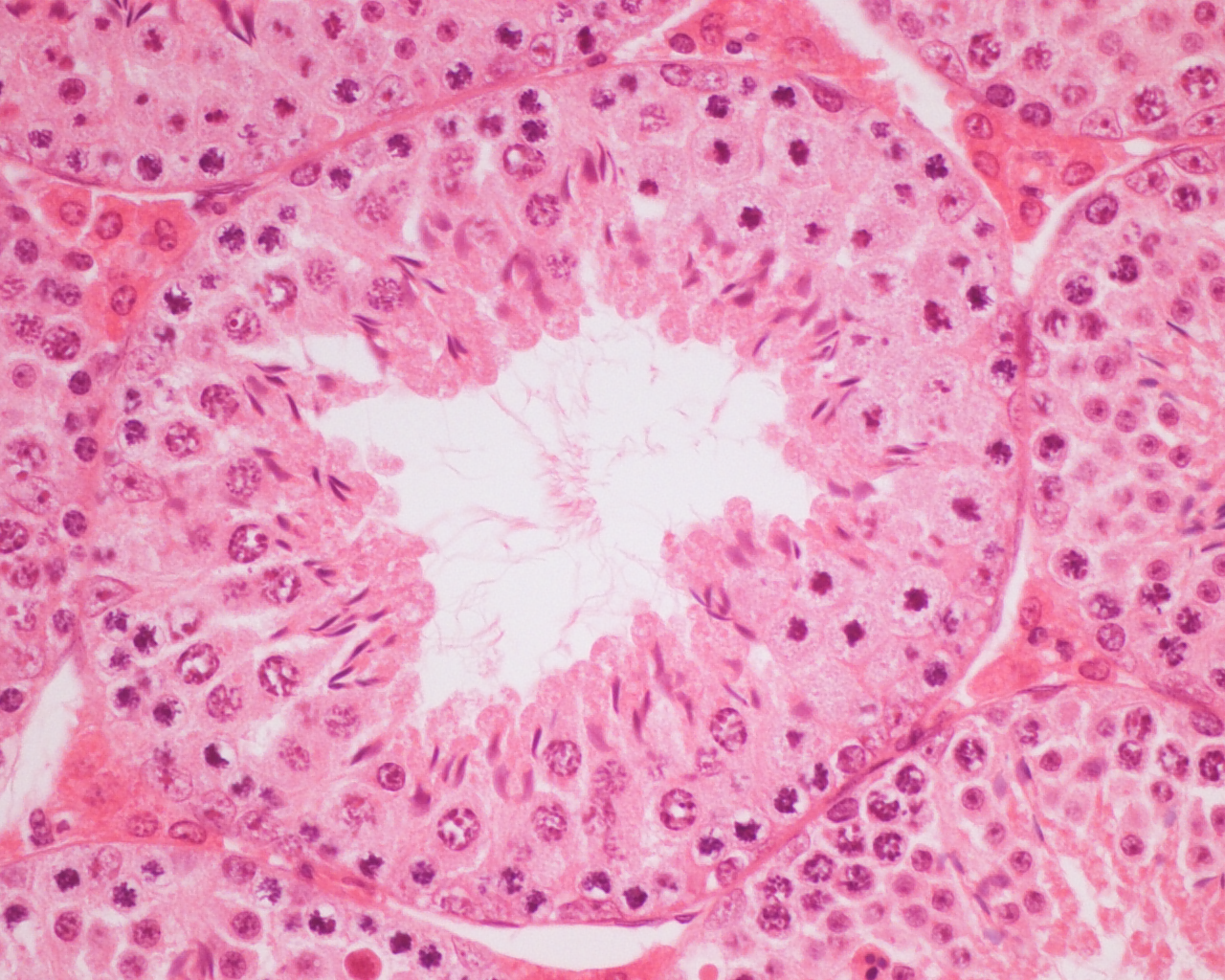

Supplement: Supplementary file 4 — Source data Fig. 1 [file 44319_2025_391_MOESM4_ESM.zip › Fig.1/G/Control-Testis-40X.tif]

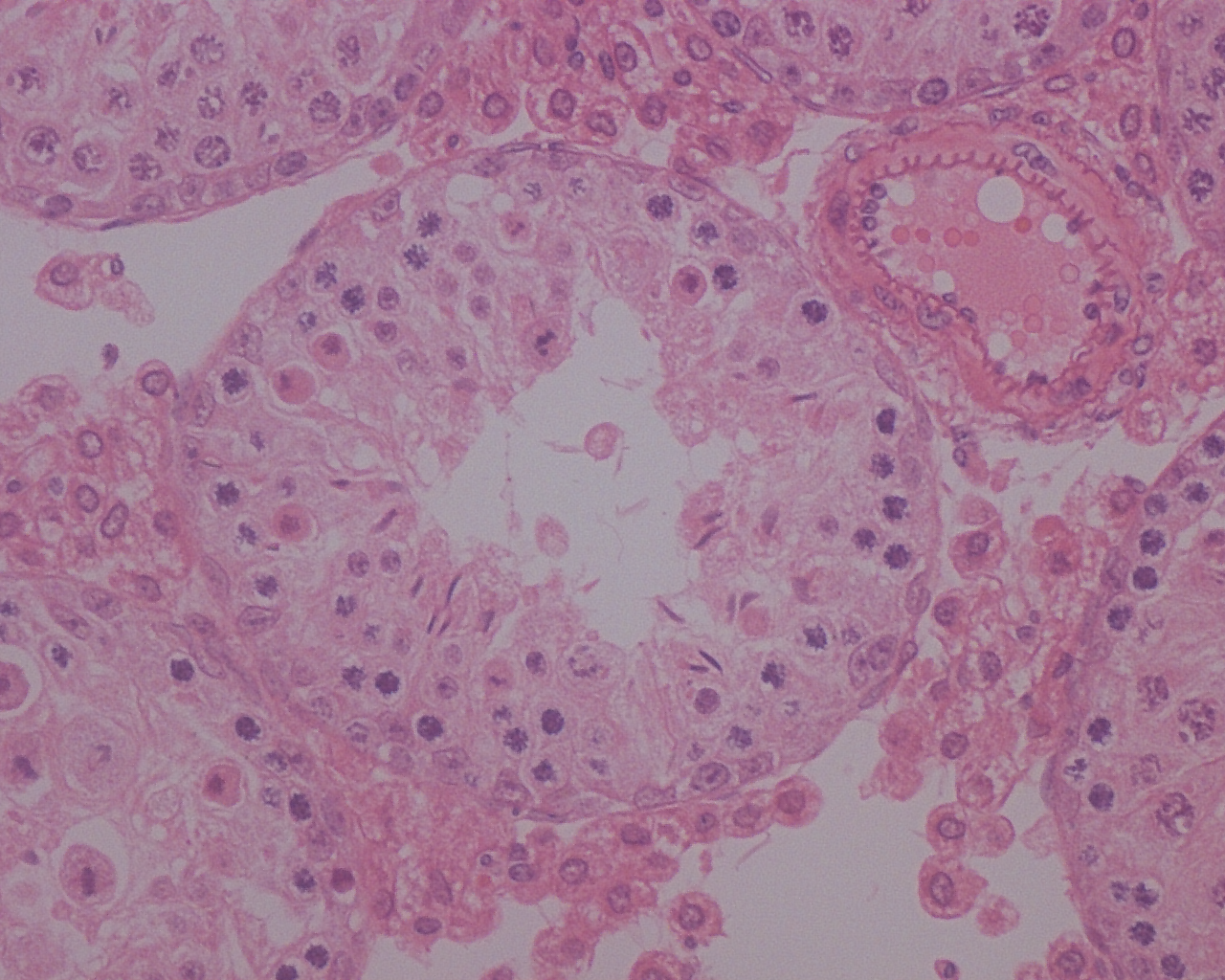

Supplement: Supplementary file 4 — Source data Fig. 1 [file 44319_2025_391_MOESM4_ESM.zip › Fig.1/G/KO-Testis-40X.tif]

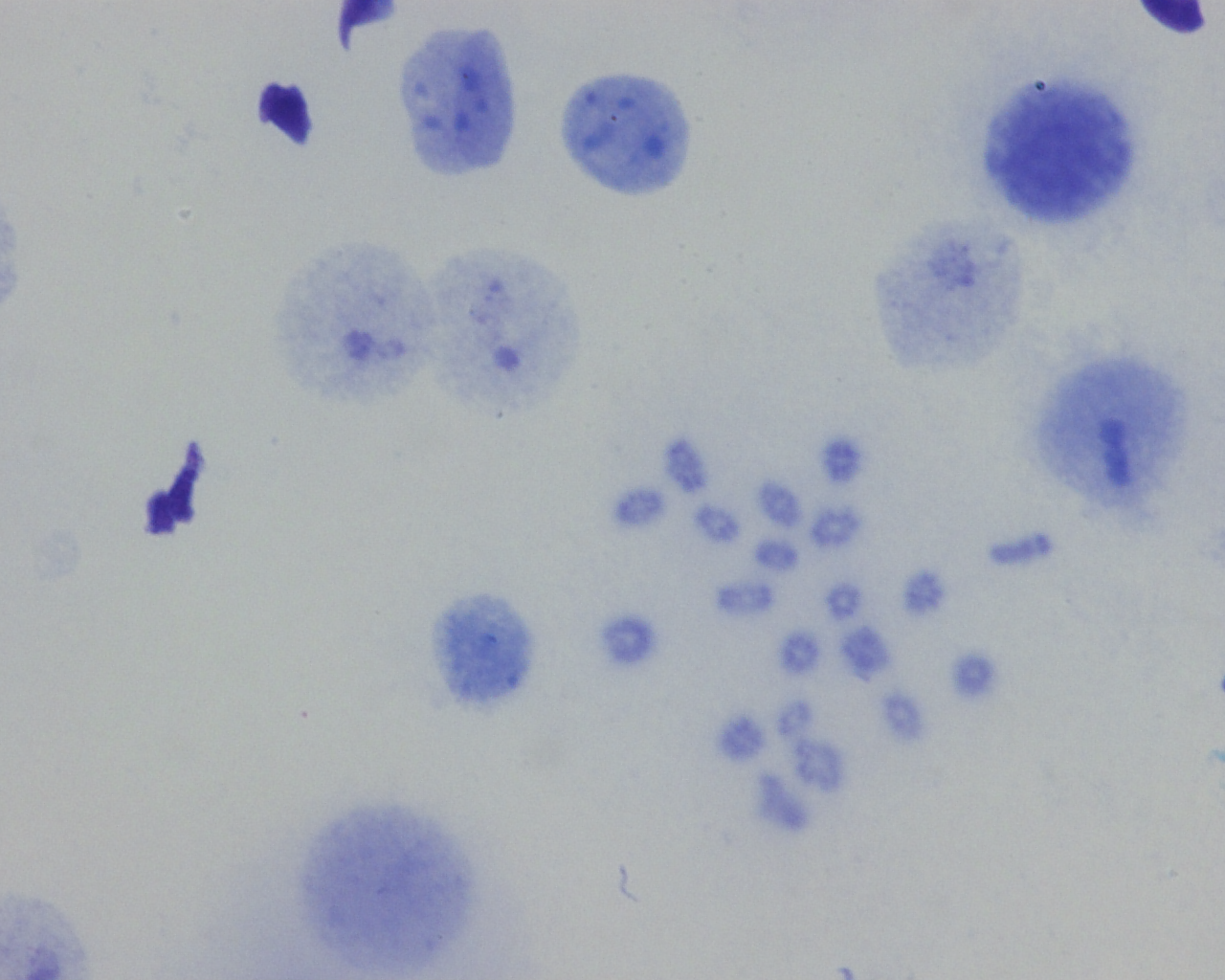

Supplement: Supplementary file 4 — Source data Fig. 1 [file 44319_2025_391_MOESM4_ESM.zip › Fig.1/H/Control.tif]

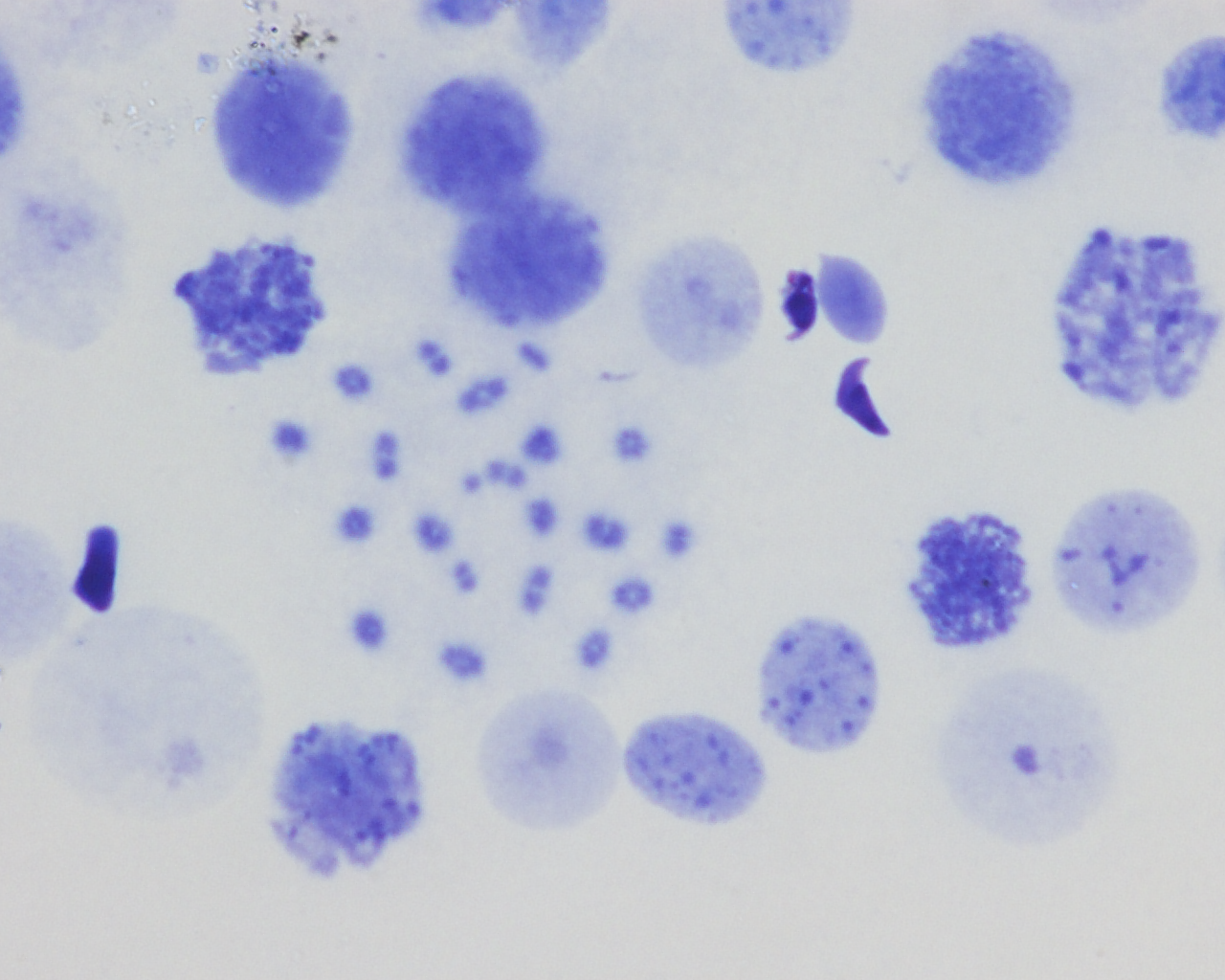

Supplement: Supplementary file 4 — Source data Fig. 1 [file 44319_2025_391_MOESM4_ESM.zip › Fig.1/H/S100PBP-KO.tif]

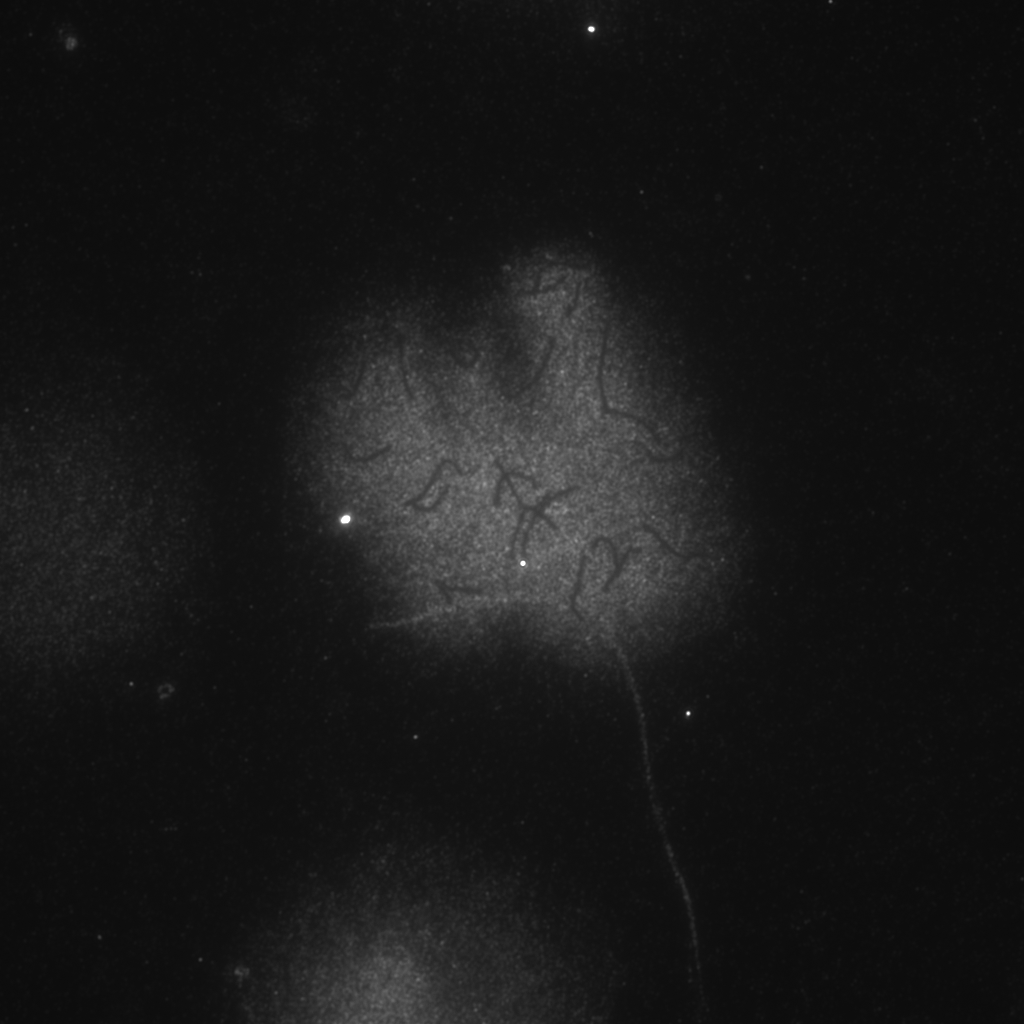

Supplement: Supplementary file 5 — Source data Fig. 2 [file 44319_2025_391_MOESM5_ESM.zip › Fig.2/A/KO/H1T.tif]

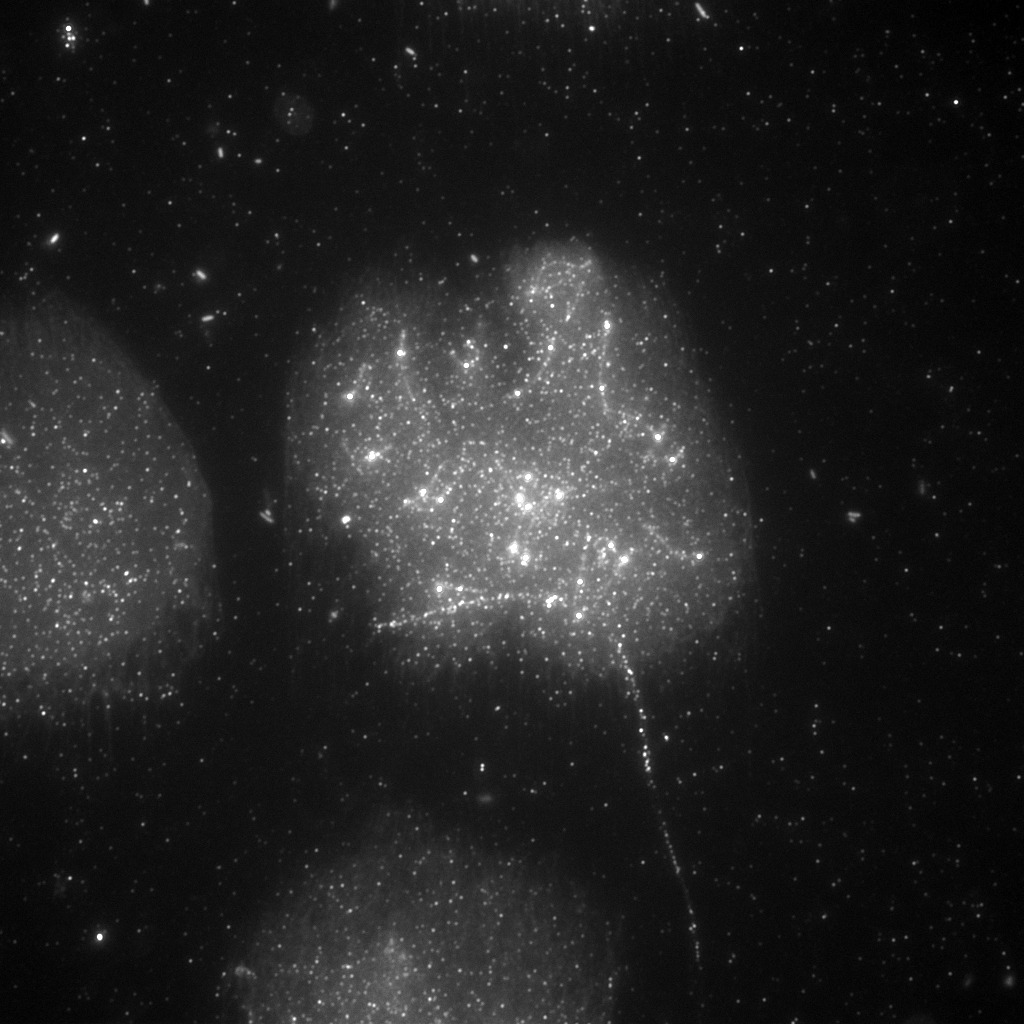

Supplement: Supplementary file 5 — Source data Fig. 2 [file 44319_2025_391_MOESM5_ESM.zip › Fig.2/A/KO/MLH1.tif]

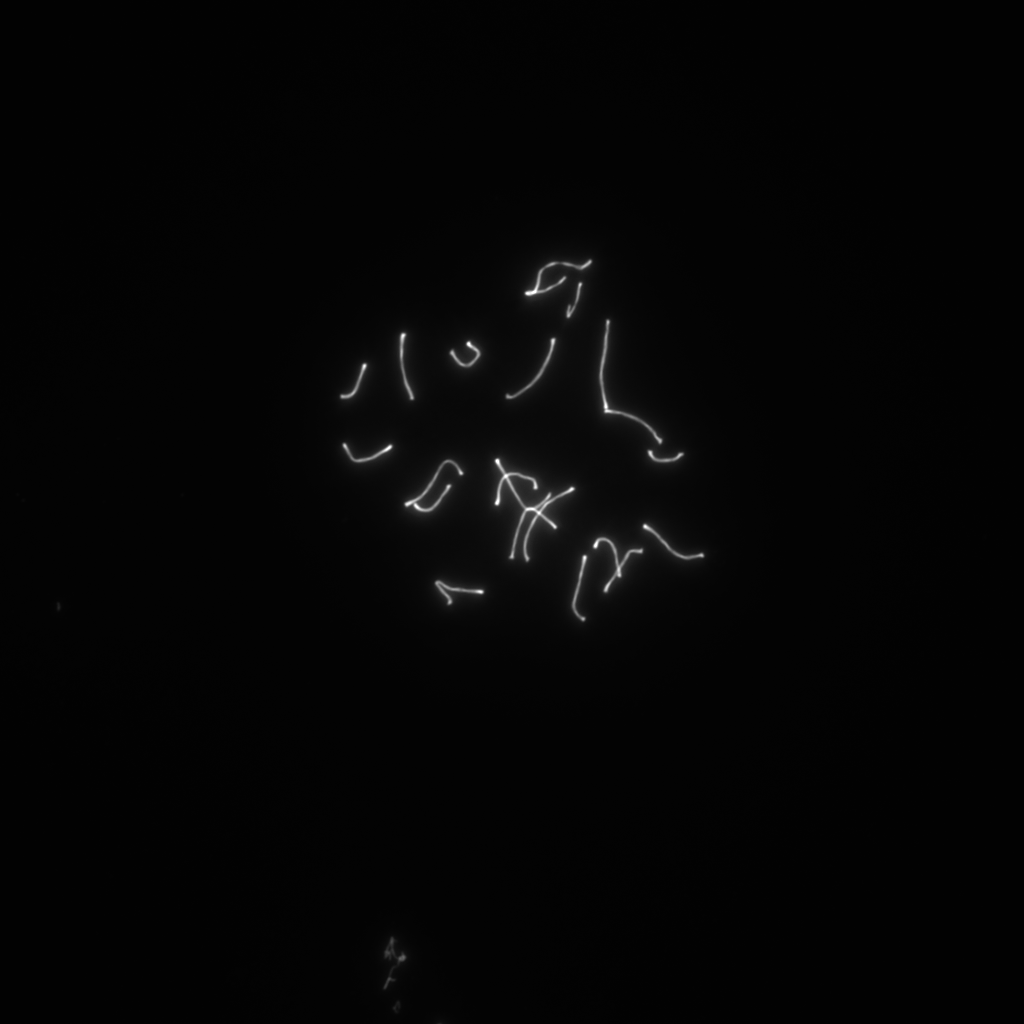

Supplement: Supplementary file 5 — Source data Fig. 2 [file 44319_2025_391_MOESM5_ESM.zip › Fig.2/A/KO/SYCP3.tif]

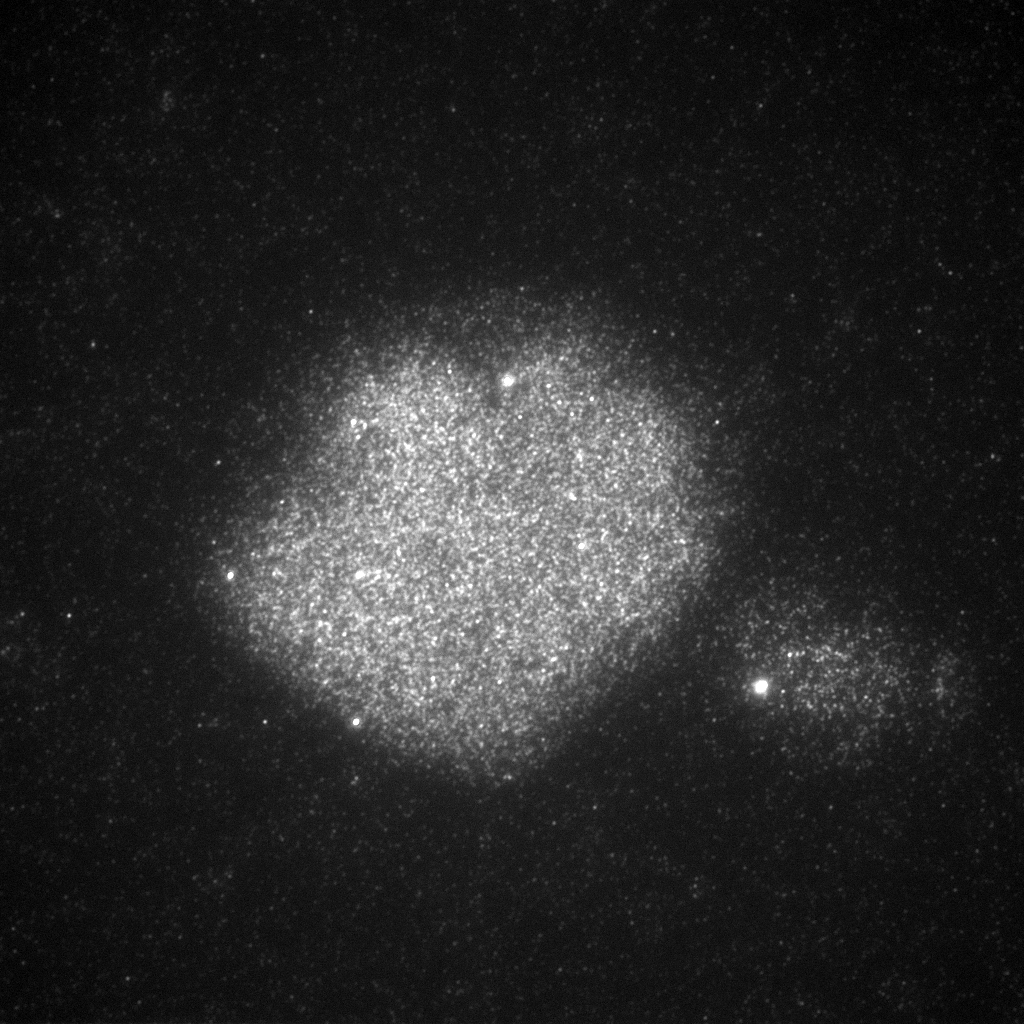

Supplement: Supplementary file 5 — Source data Fig. 2 [file 44319_2025_391_MOESM5_ESM.zip › Fig.2/A/WT/H1T.tif]

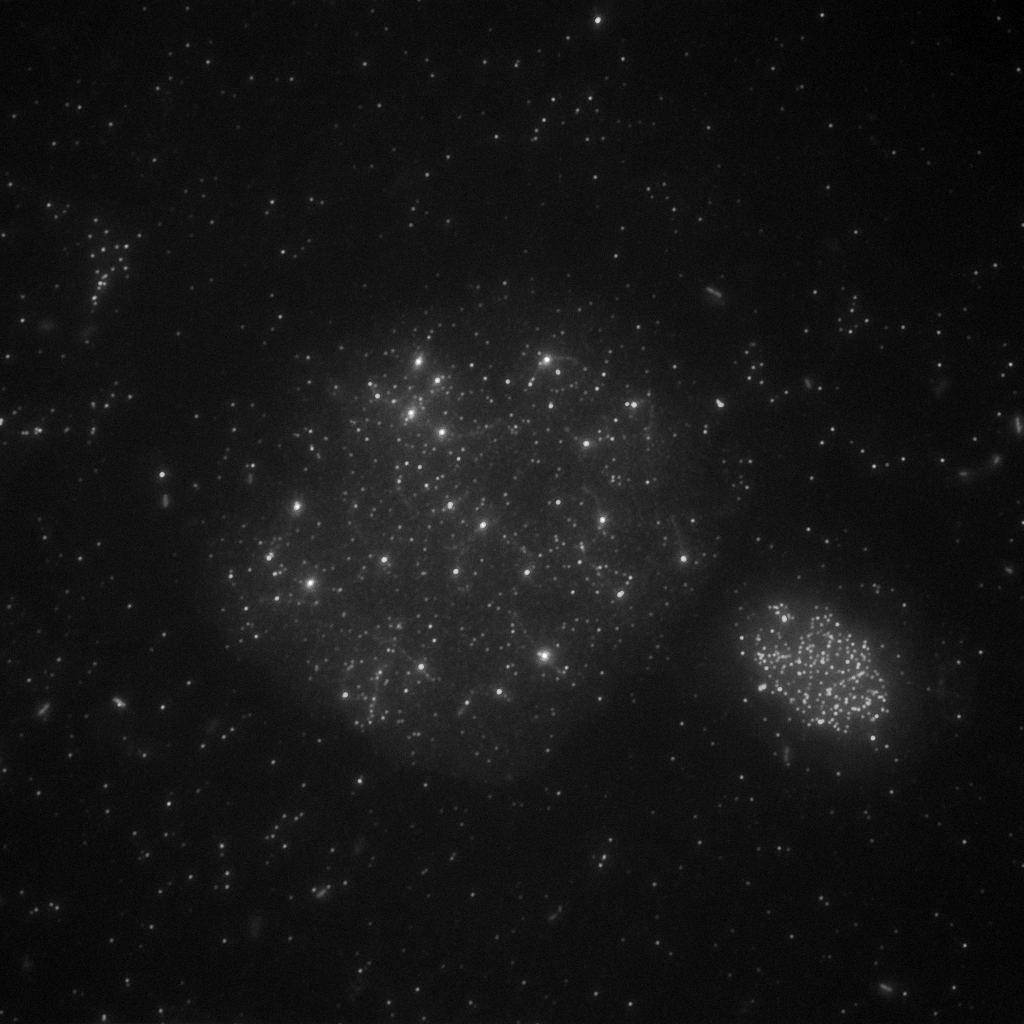

Supplement: Supplementary file 5 — Source data Fig. 2 [file 44319_2025_391_MOESM5_ESM.zip › Fig.2/A/WT/MLH1.tif]

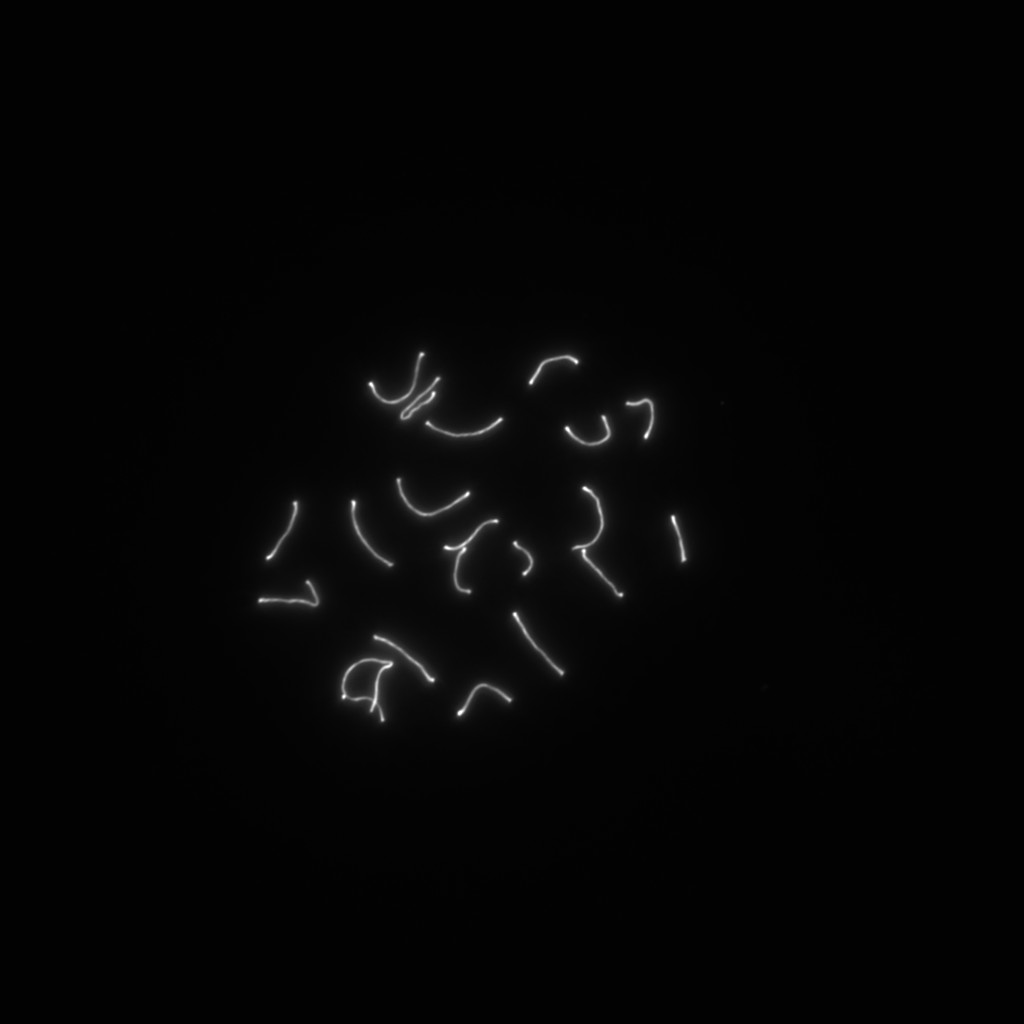

Supplement: Supplementary file 5 — Source data Fig. 2 [file 44319_2025_391_MOESM5_ESM.zip › Fig.2/A/WT/SYCP3.tif]

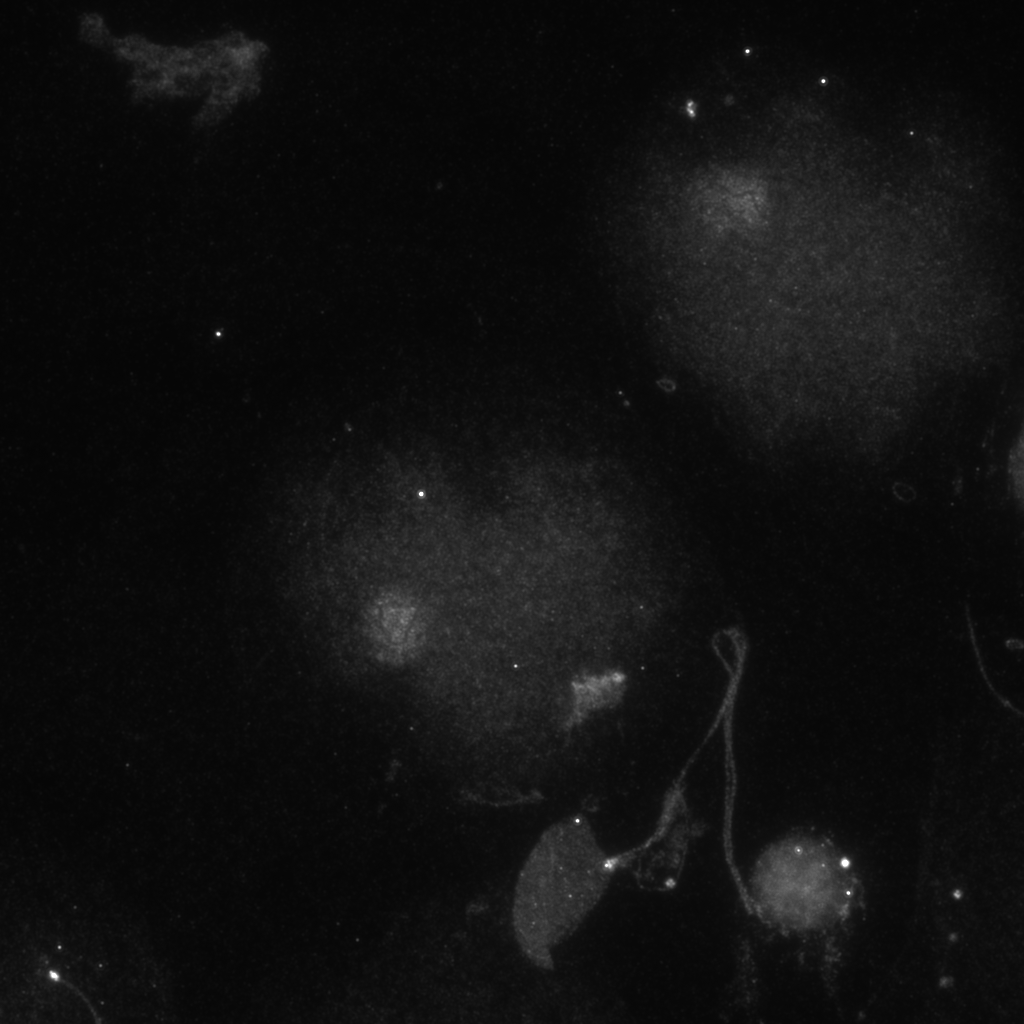

Supplement: Supplementary file 5 — Source data Fig. 2 [file 44319_2025_391_MOESM5_ESM.zip › Fig.2/E/KO/H1T.tif]

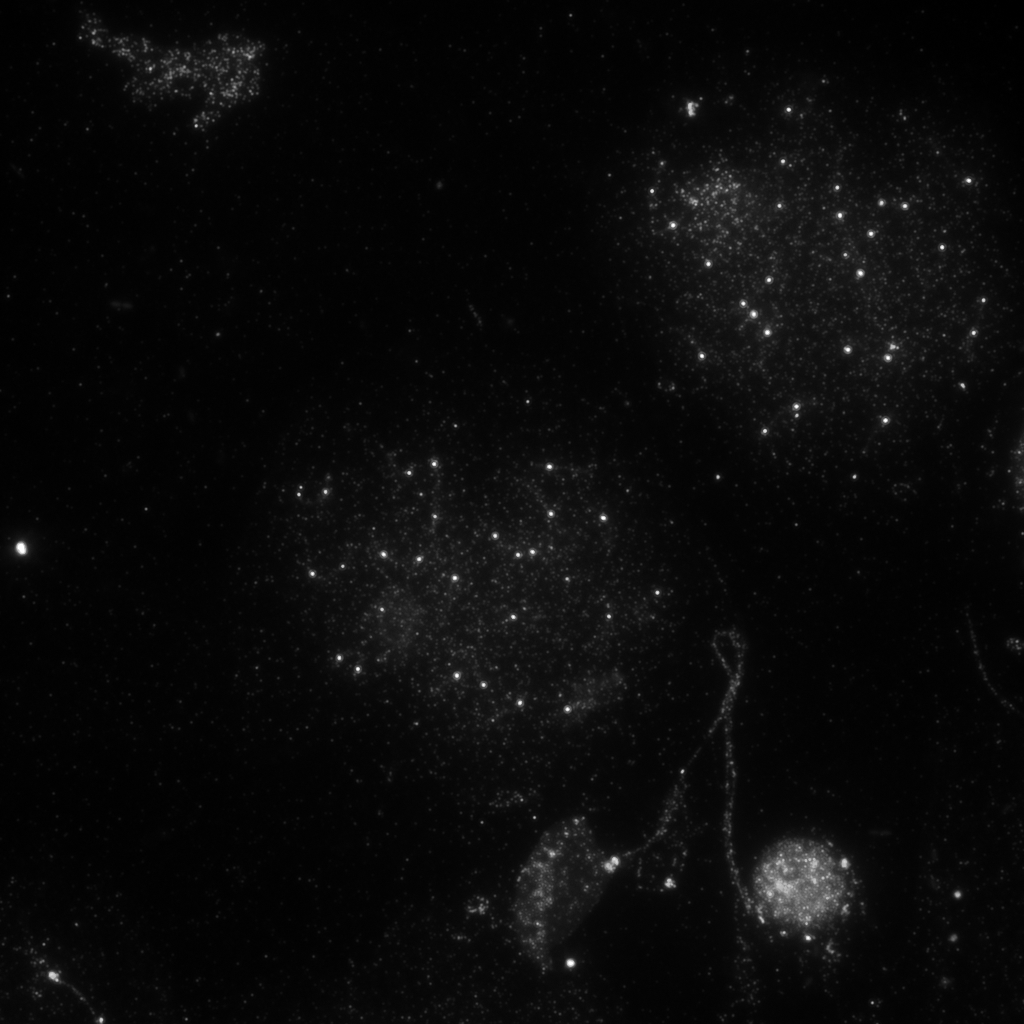

Supplement: Supplementary file 5 — Source data Fig. 2 [file 44319_2025_391_MOESM5_ESM.zip › Fig.2/E/KO/MLH3.tif]

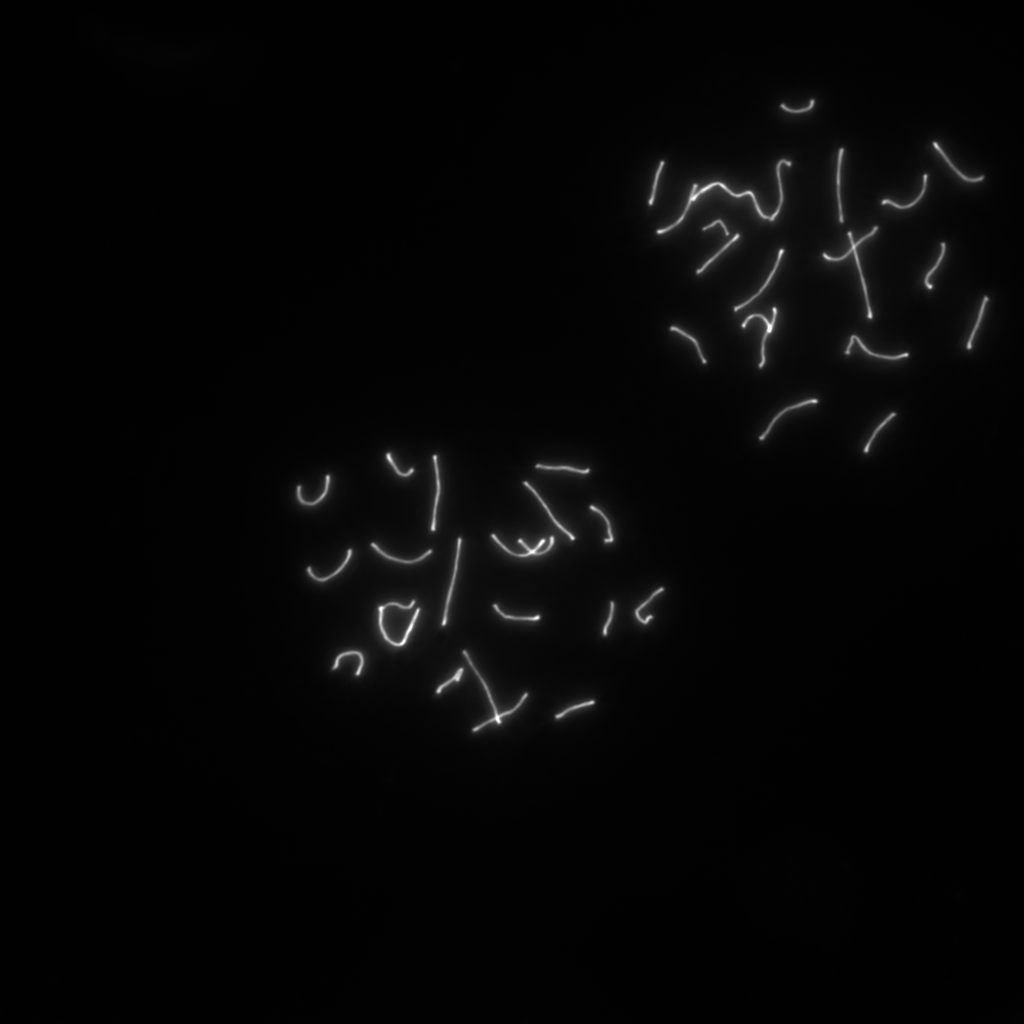

Supplement: Supplementary file 5 — Source data Fig. 2 [file 44319_2025_391_MOESM5_ESM.zip › Fig.2/E/KO/SYCP3.tif]

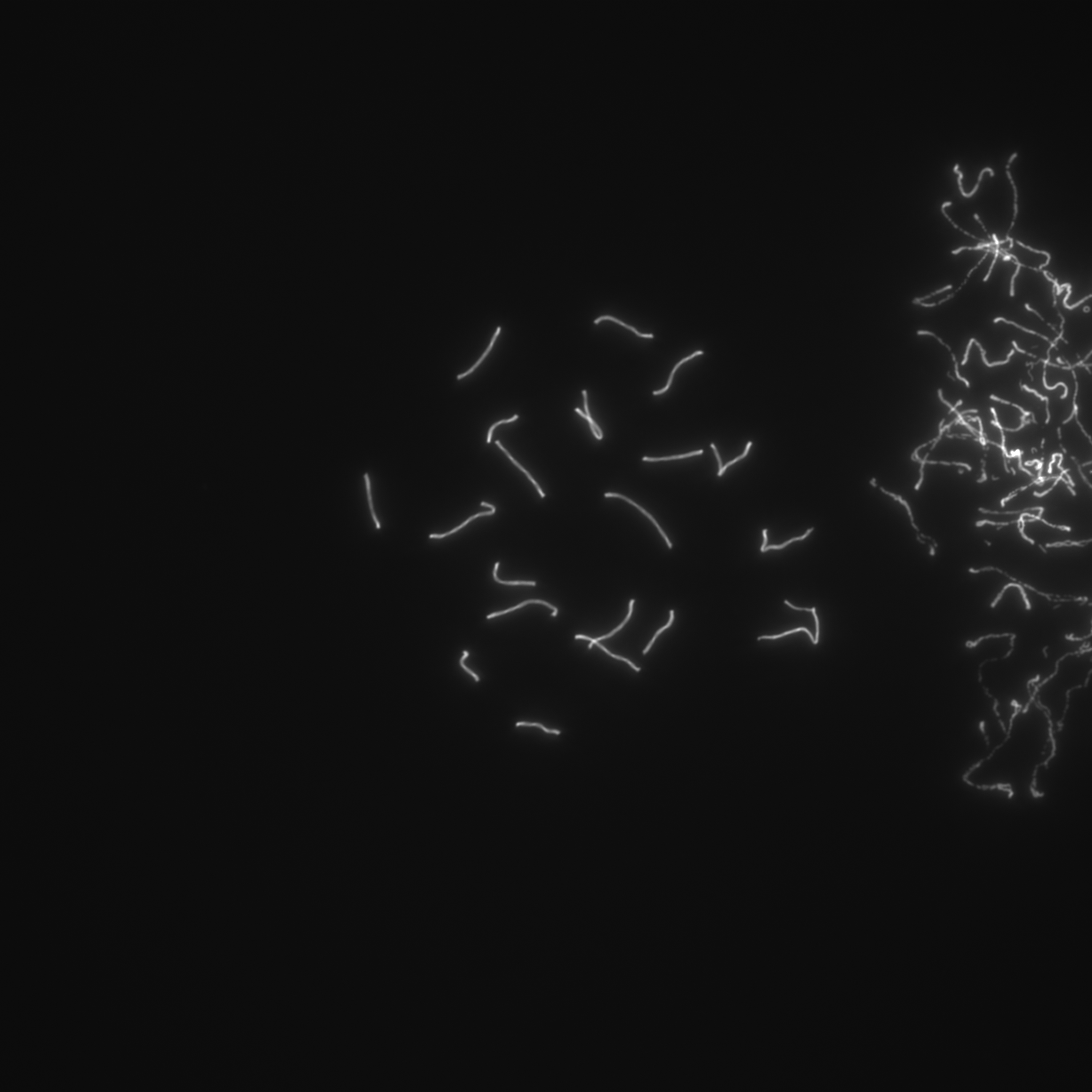

Supplement: Supplementary file 5 — Source data Fig. 2 [file 44319_2025_391_MOESM5_ESM.zip › Fig.2/E/WT/CP3.tif]

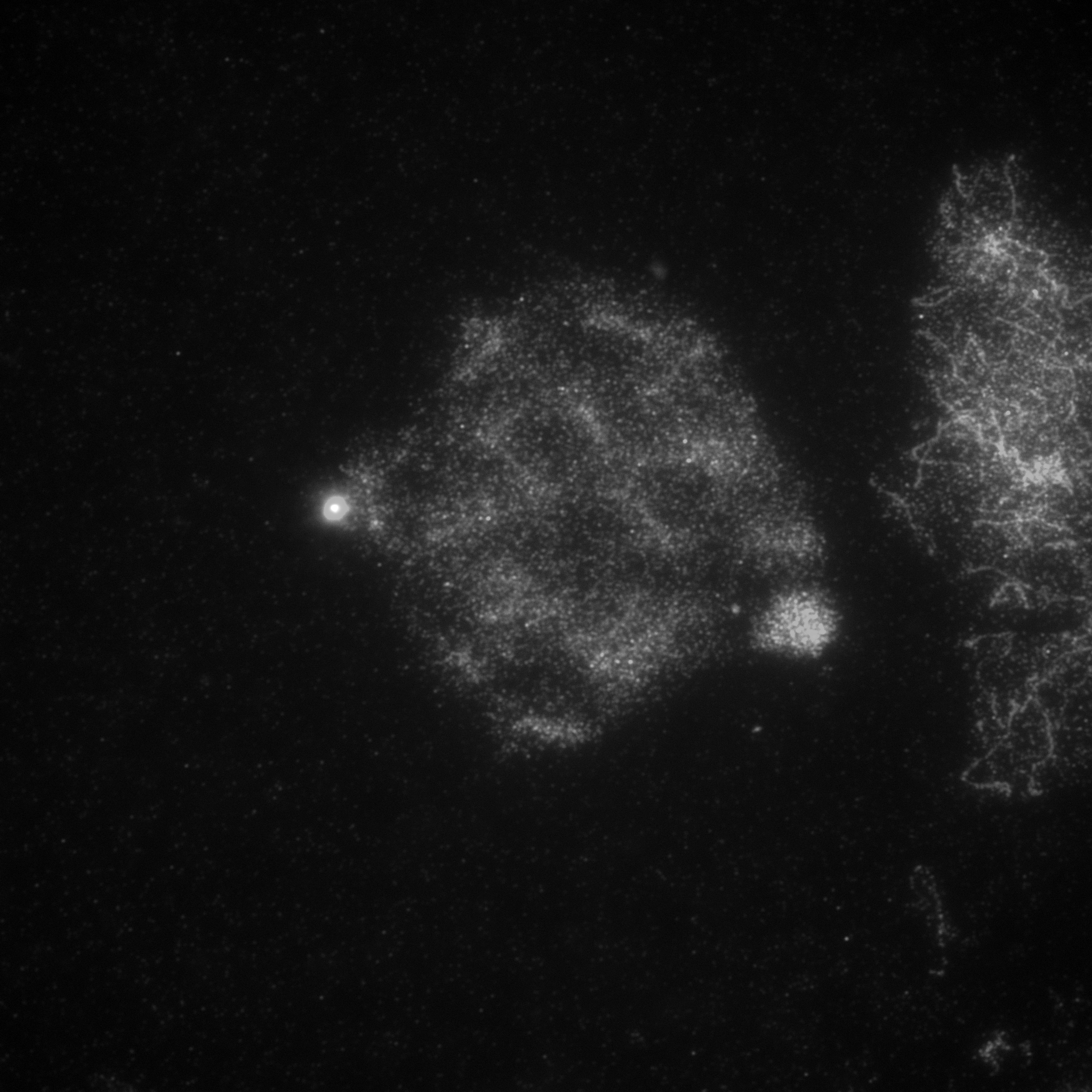

Supplement: Supplementary file 5 — Source data Fig. 2 [file 44319_2025_391_MOESM5_ESM.zip › Fig.2/E/WT/H1T.tif]

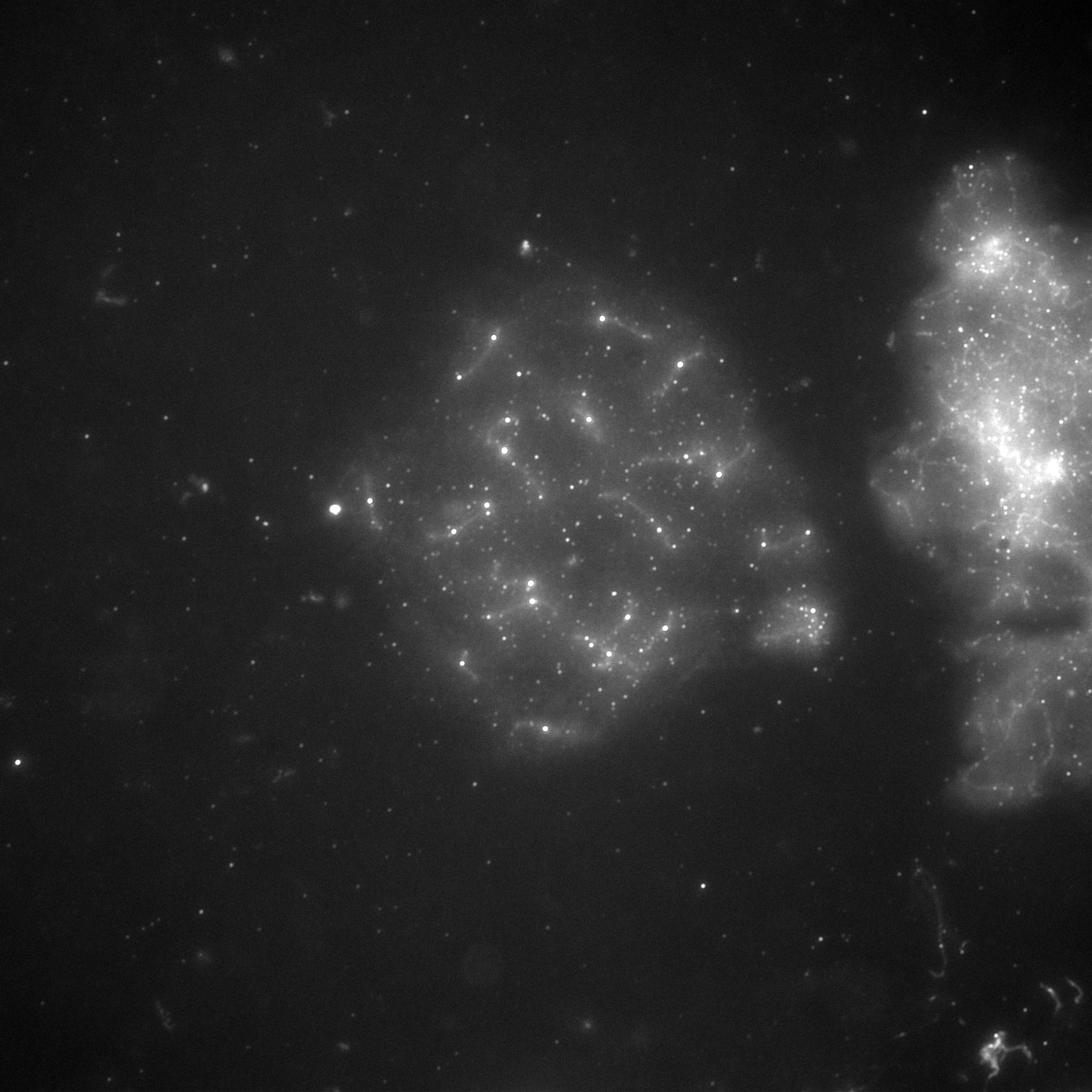

Supplement: Supplementary file 5 — Source data Fig. 2 [file 44319_2025_391_MOESM5_ESM.zip › Fig.2/E/WT/MLH3.tif]

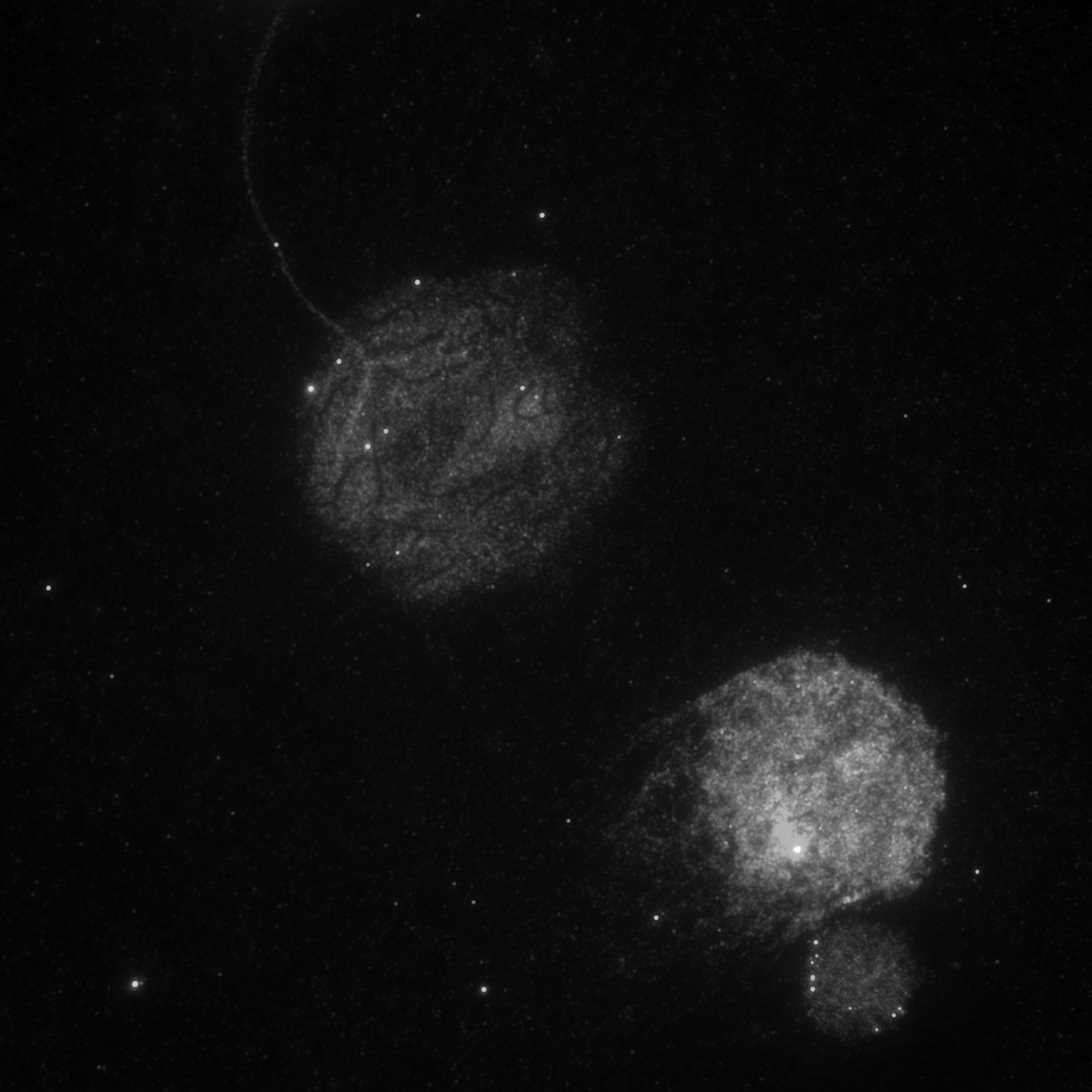

Supplement: Supplementary file 6 — Source data Fig. 3 [file 44319_2025_391_MOESM6_ESM.zip › Fig.3/A/Control/EP-H1T.tif]

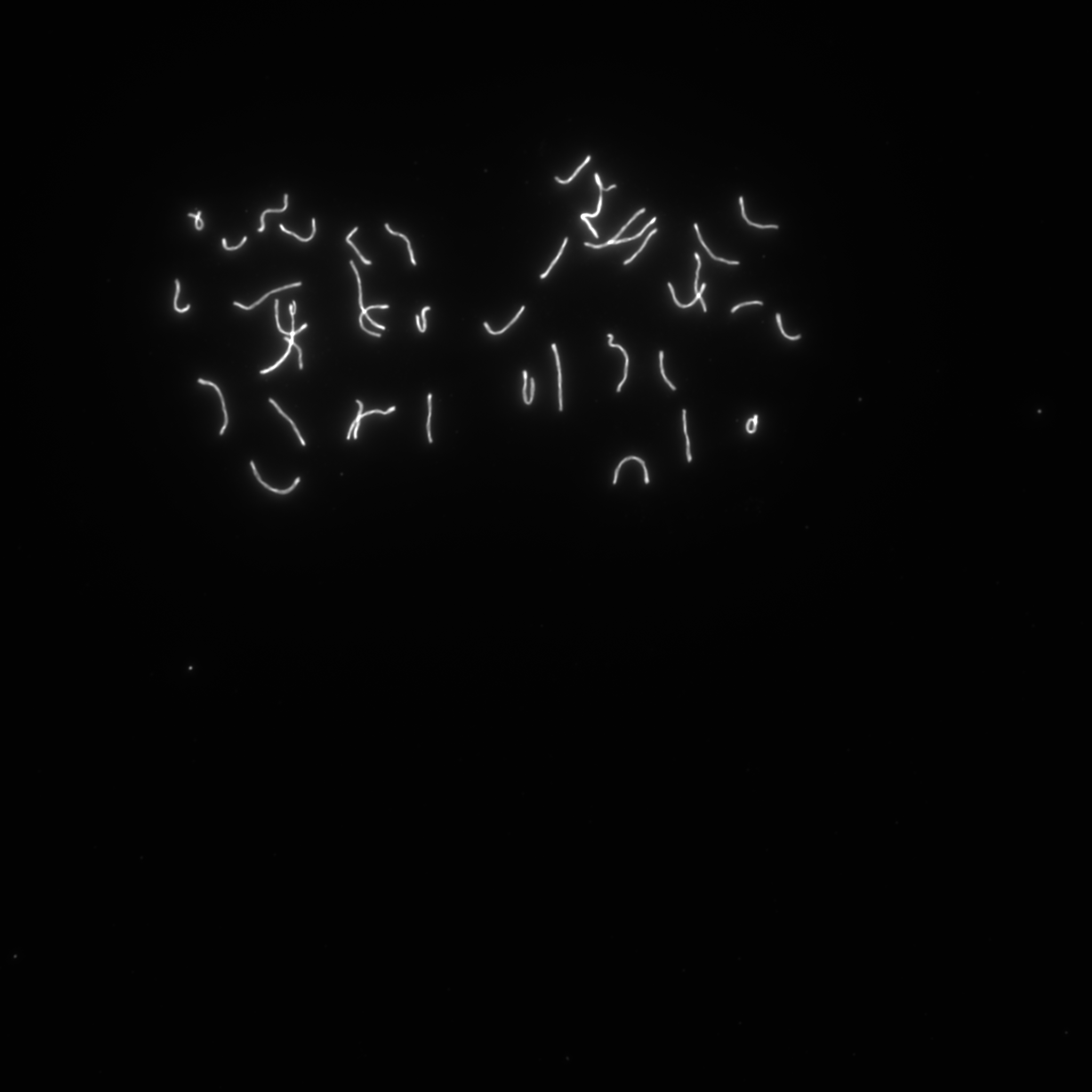

Supplement: Supplementary file 6 — Source data Fig. 3 [file 44319_2025_391_MOESM6_ESM.zip › Fig.3/A/Control/EP-SYCP3.tif]

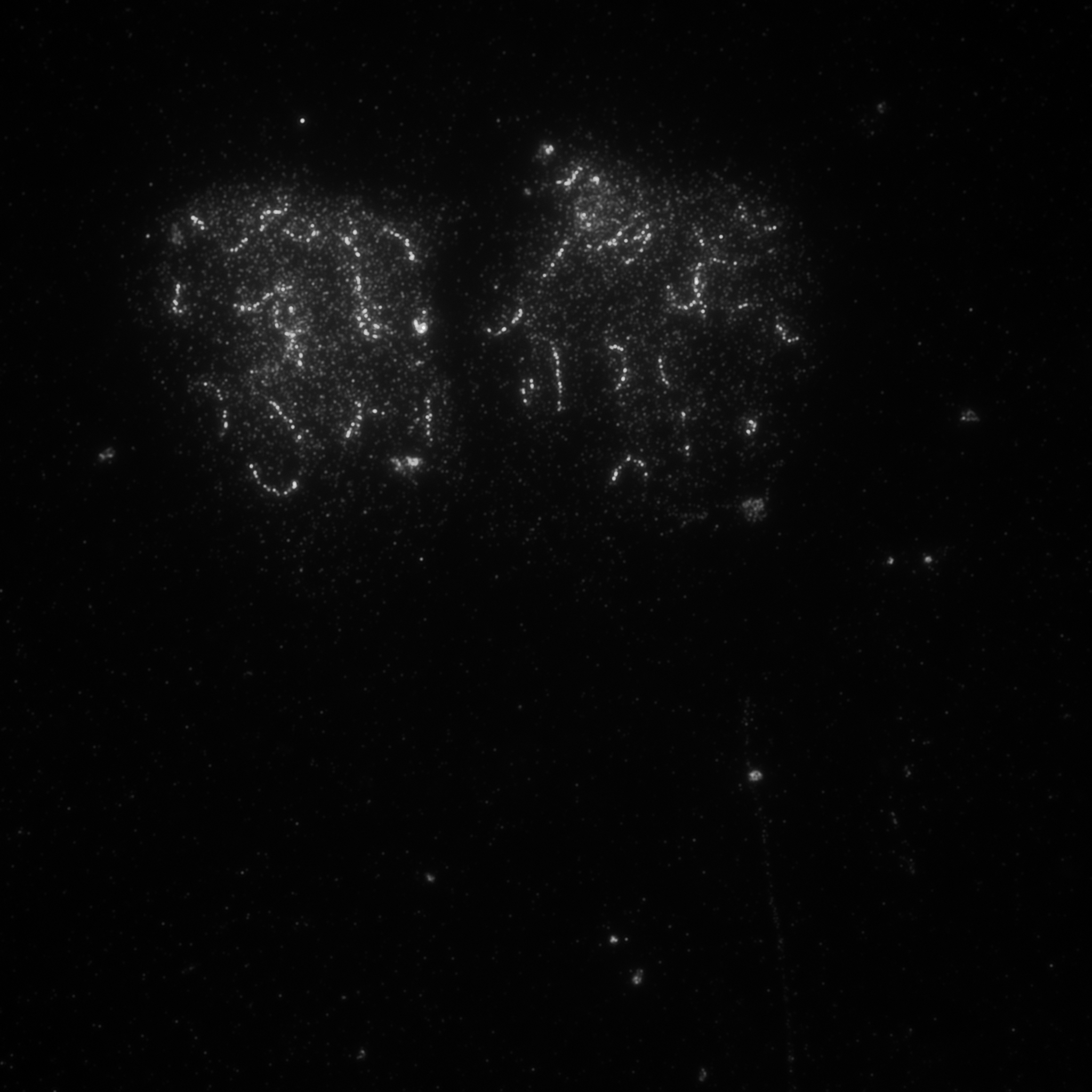

Supplement: Supplementary file 6 — Source data Fig. 3 [file 44319_2025_391_MOESM6_ESM.zip › Fig.3/A/Control/EP-TEX11.tif]

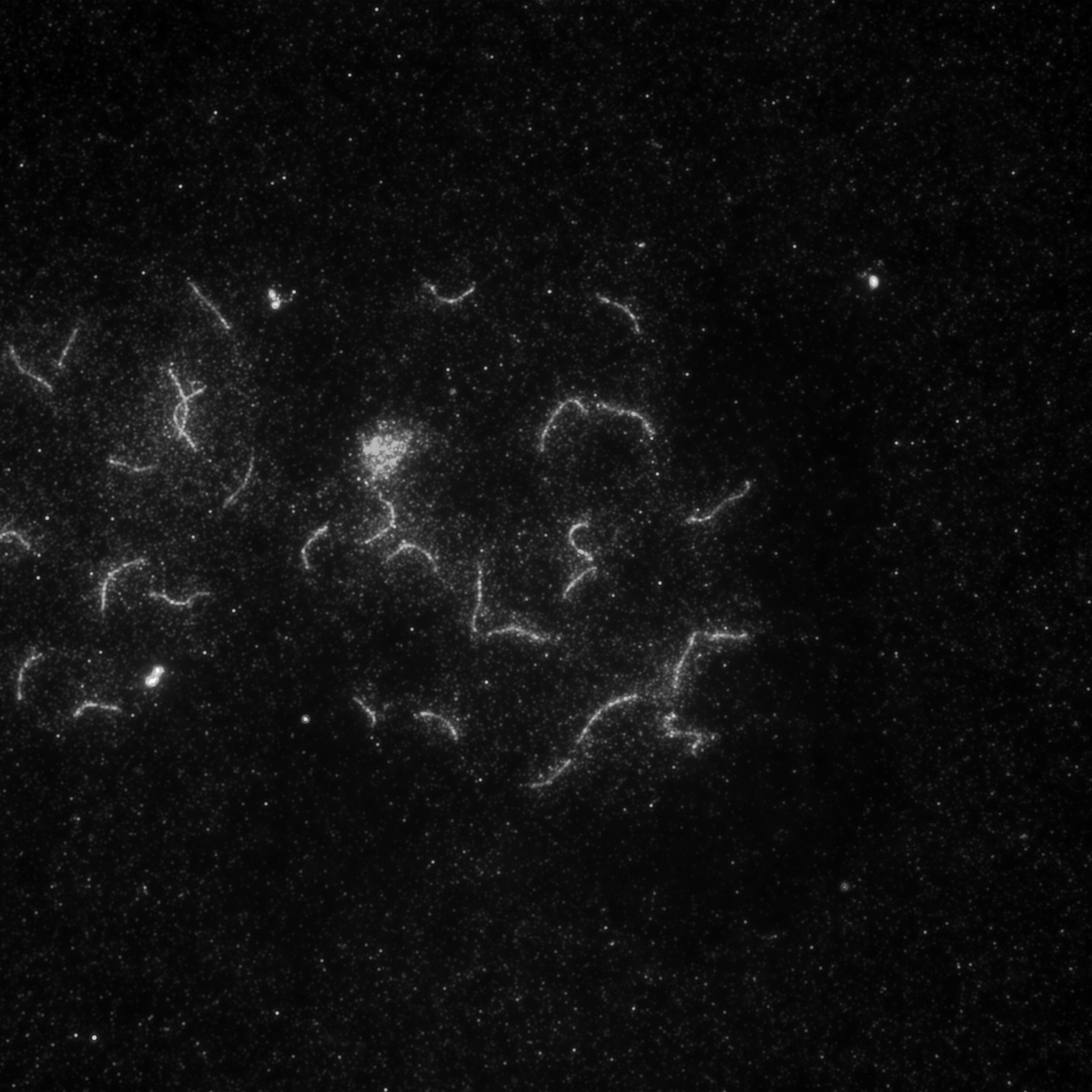

Supplement: Supplementary file 6 — Source data Fig. 3 [file 44319_2025_391_MOESM6_ESM.zip › Fig.3/D/Control/EP-H1T.tif]

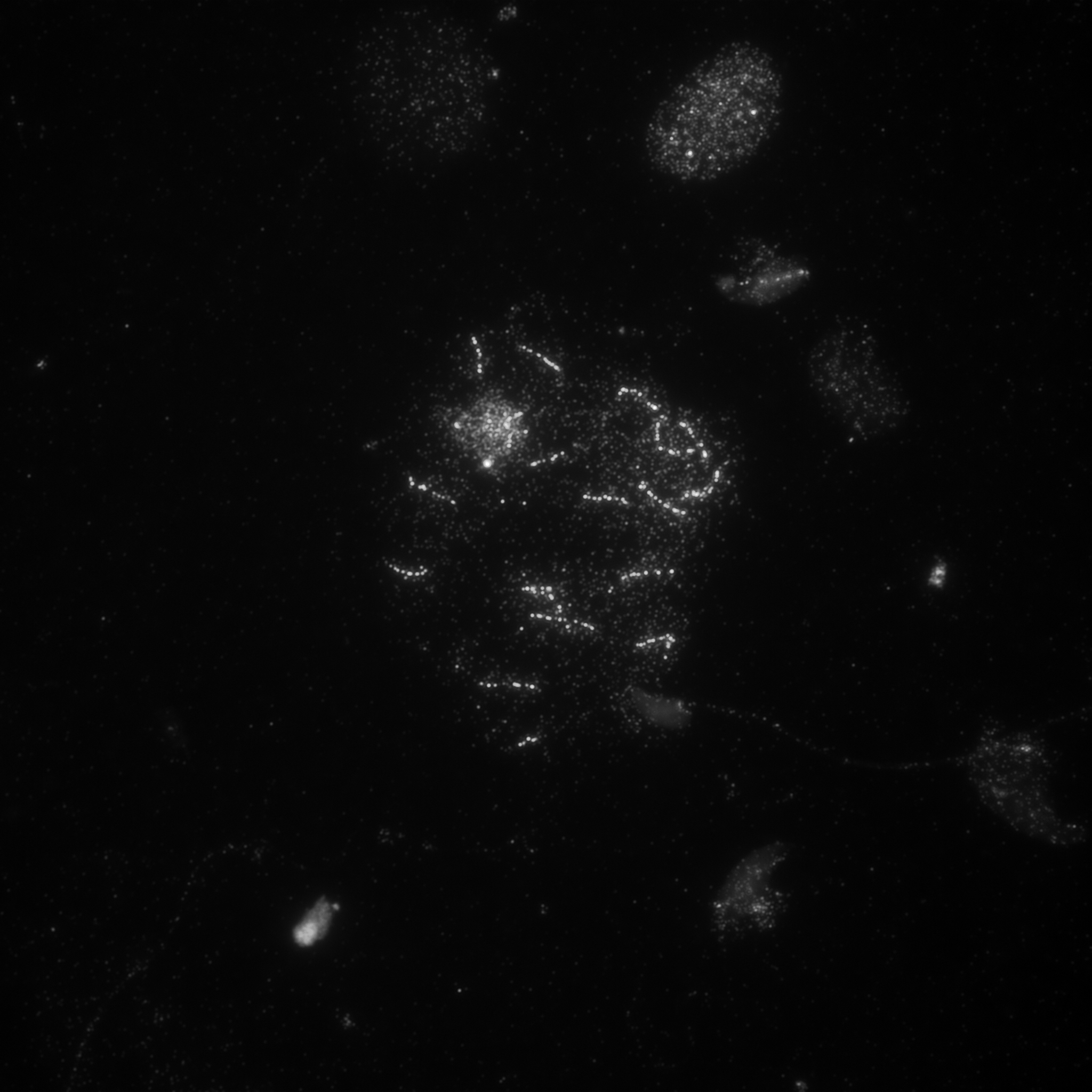

Supplement: Supplementary file 6 — Source data Fig. 3 [file 44319_2025_391_MOESM6_ESM.zip › Fig.3/D/Control/EP-M1AP.tif]

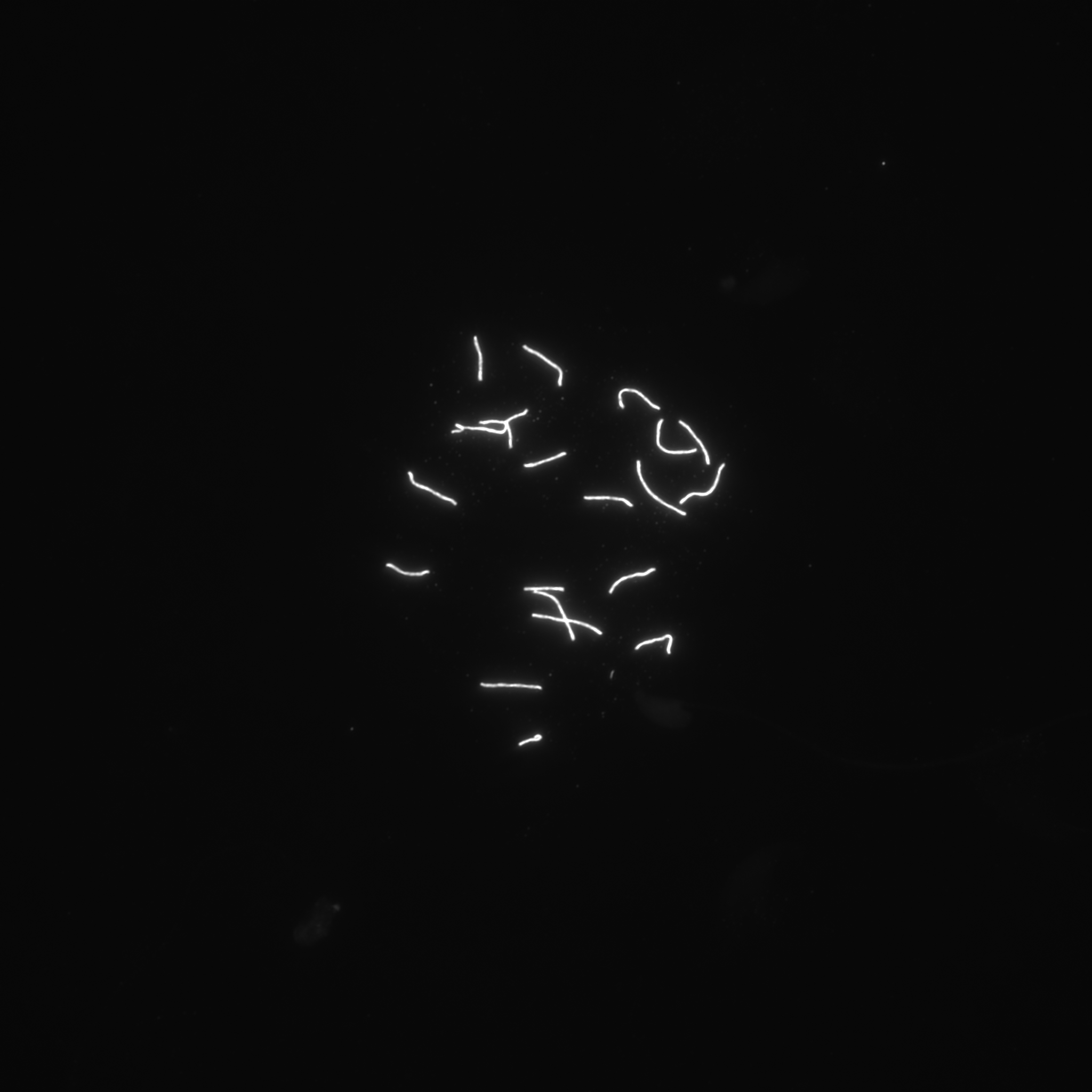

Supplement: Supplementary file 6 — Source data Fig. 3 [file 44319_2025_391_MOESM6_ESM.zip › Fig.3/D/Control/EP-SYCP3.tif]

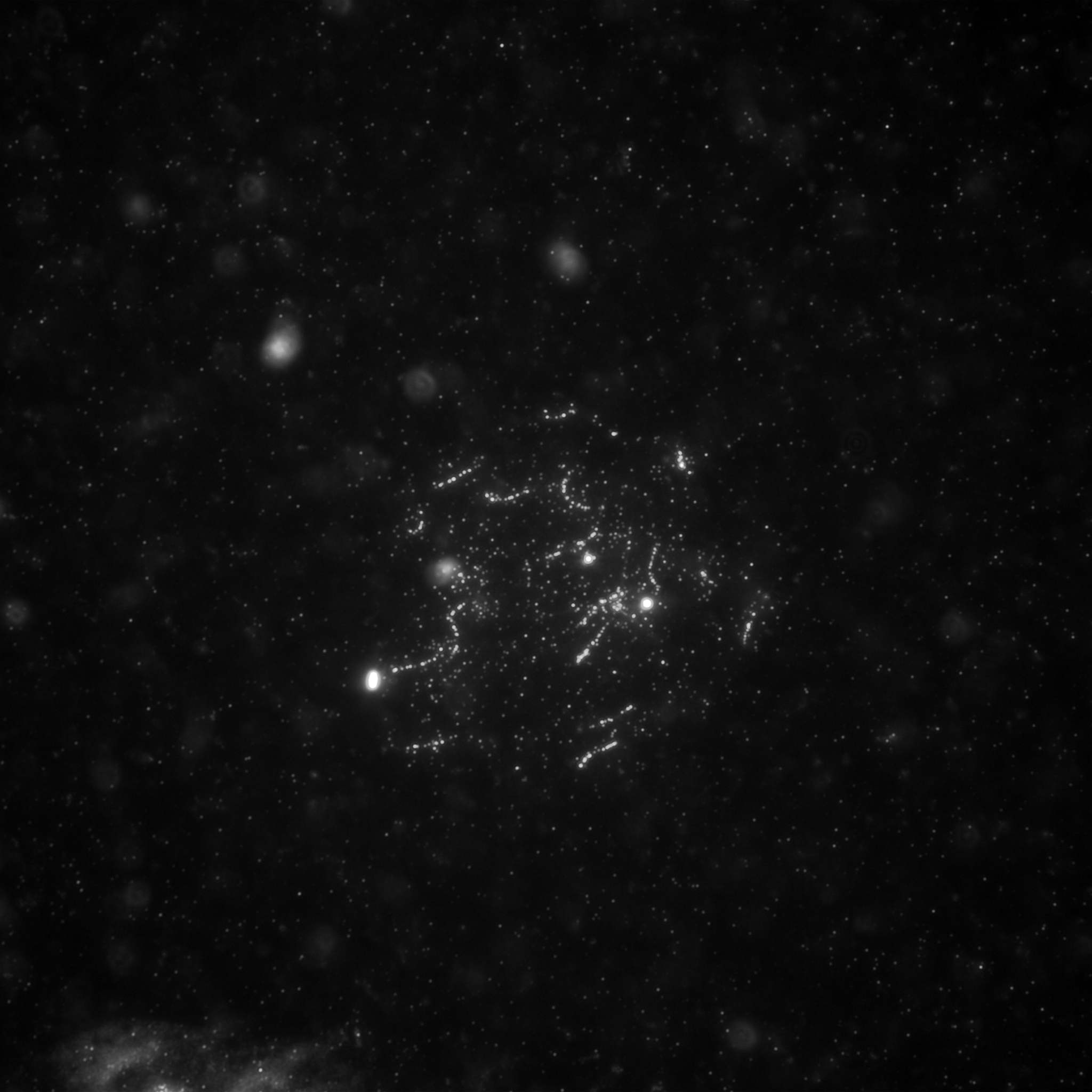

Supplement: Supplementary file 6 — Source data Fig. 3 [file 44319_2025_391_MOESM6_ESM.zip › Fig.3/D/Control/LZ-M1AP.tif]

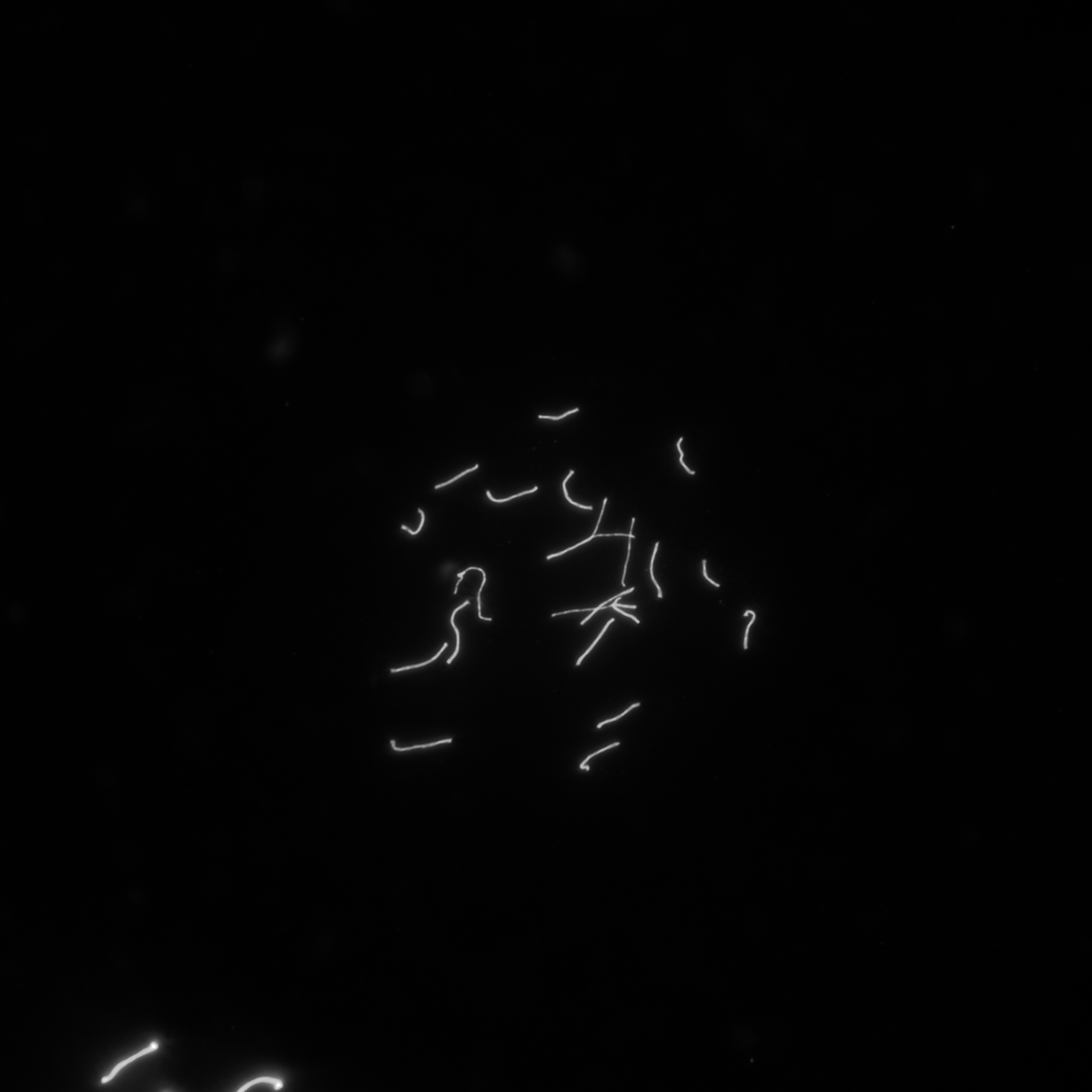

Supplement: Supplementary file 6 — Source data Fig. 3 [file 44319_2025_391_MOESM6_ESM.zip › Fig.3/D/Control/LZ-SYCP3.tif]

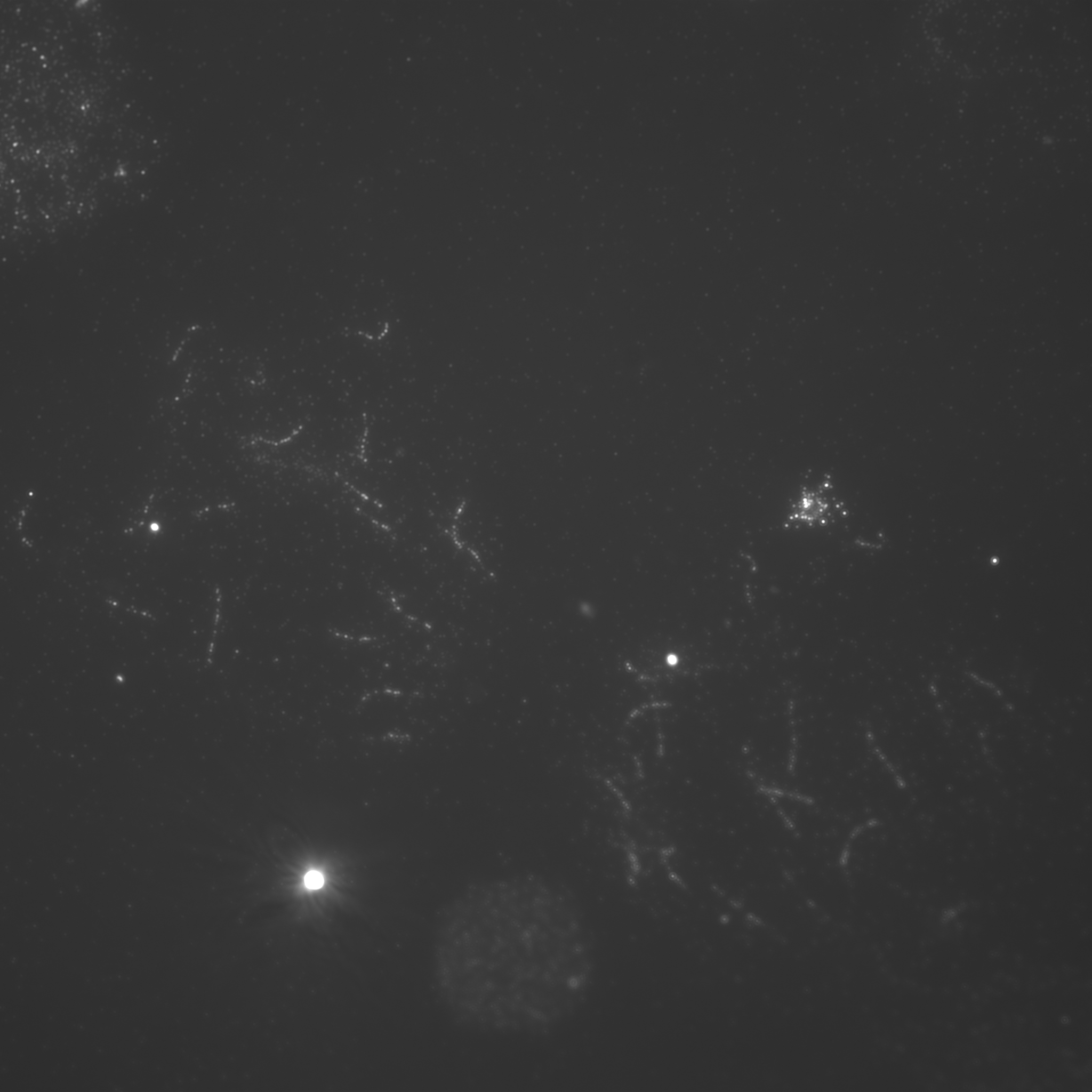

Supplement: Supplementary file 6 — Source data Fig. 3 [file 44319_2025_391_MOESM6_ESM.zip › Fig.3/D/KO/LZ-M1AP.tif]

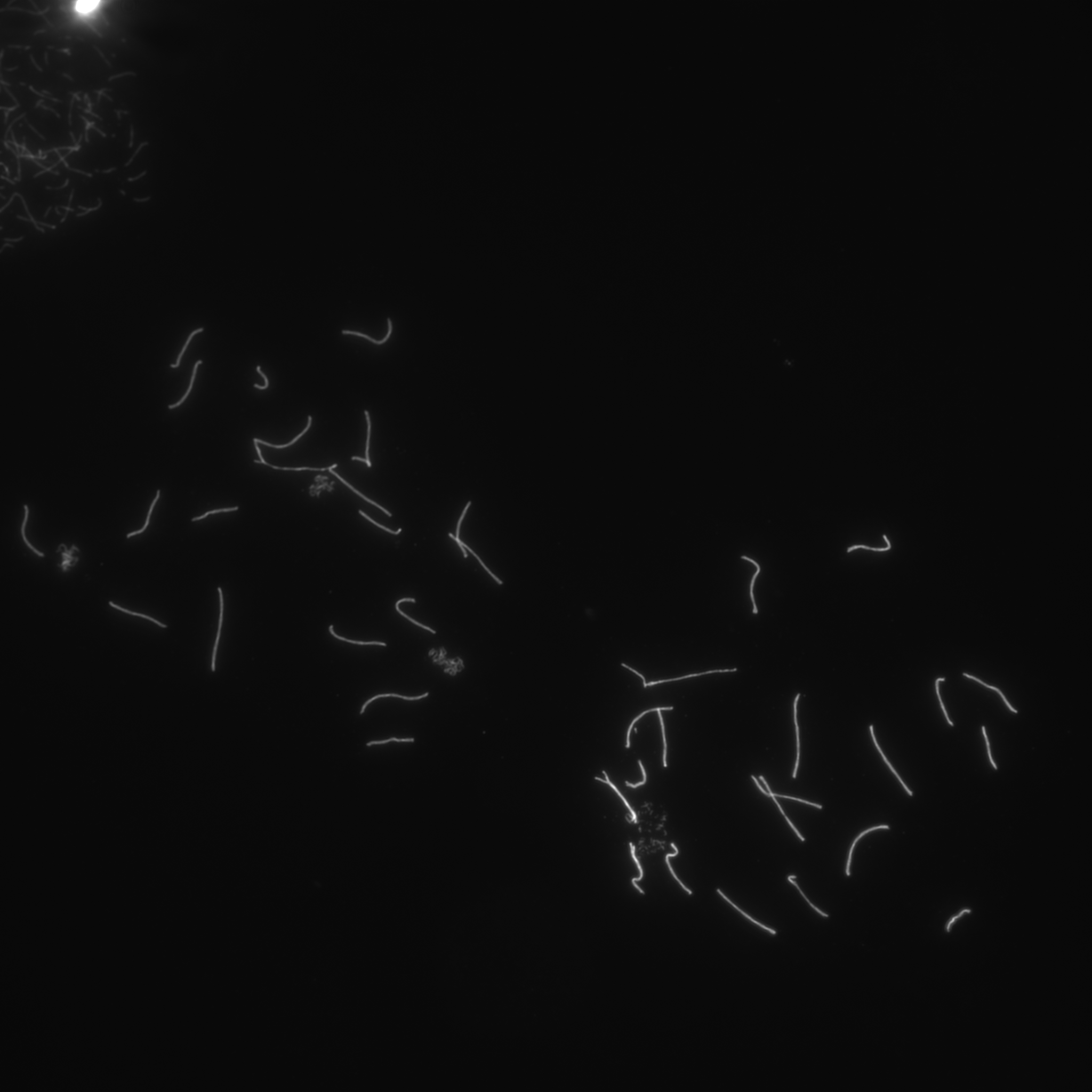

Supplement: Supplementary file 6 — Source data Fig. 3 [file 44319_2025_391_MOESM6_ESM.zip › Fig.3/D/KO/LZ-SYCP3.tif]

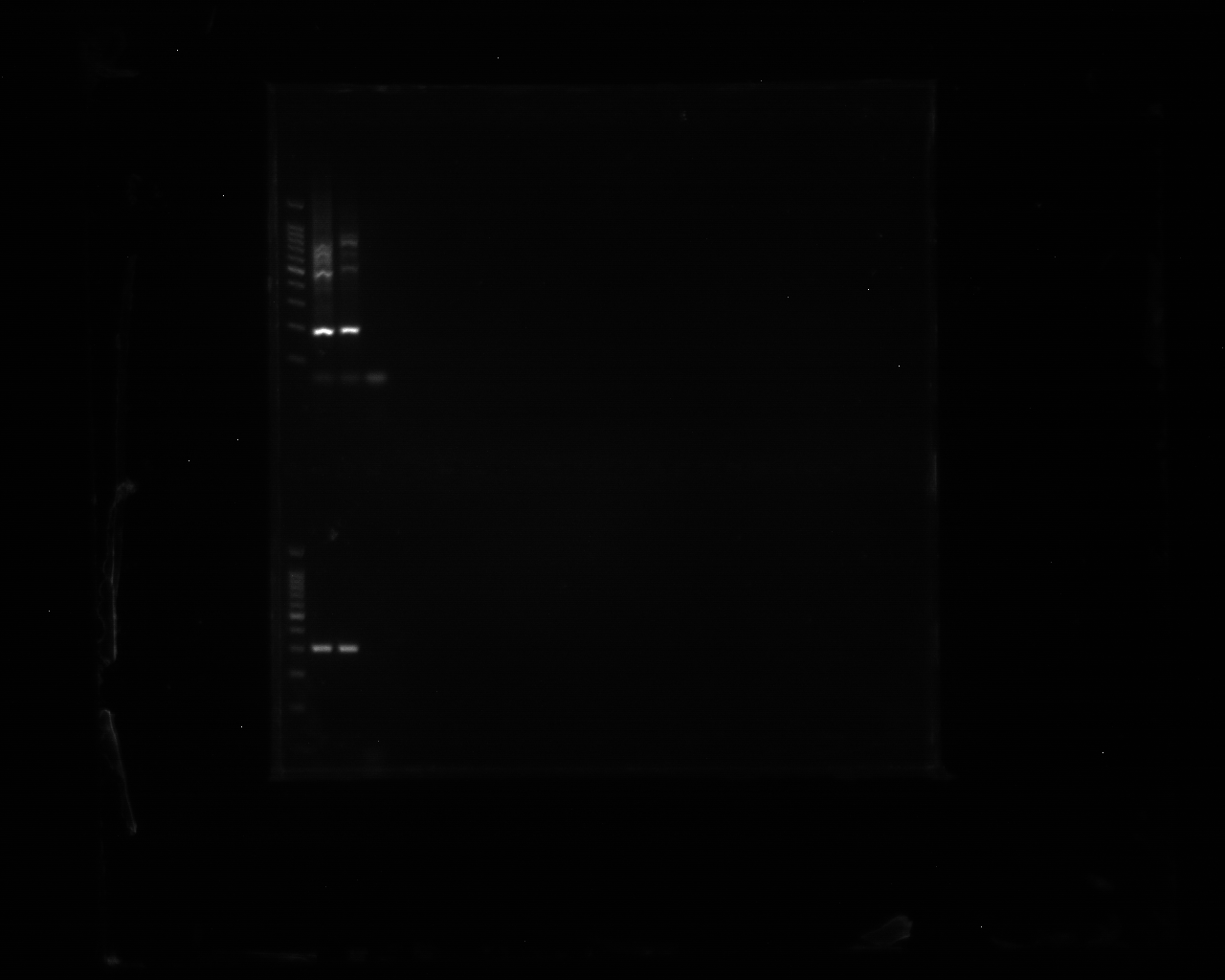

Supplement: Supplementary file 7 — Source data Fig. 4 [file 44319_2025_391_MOESM7_ESM.zip › Fig.4/A/S100pbp-Testis-ovary-H2O/32cycle,act-25cycle.Tif]

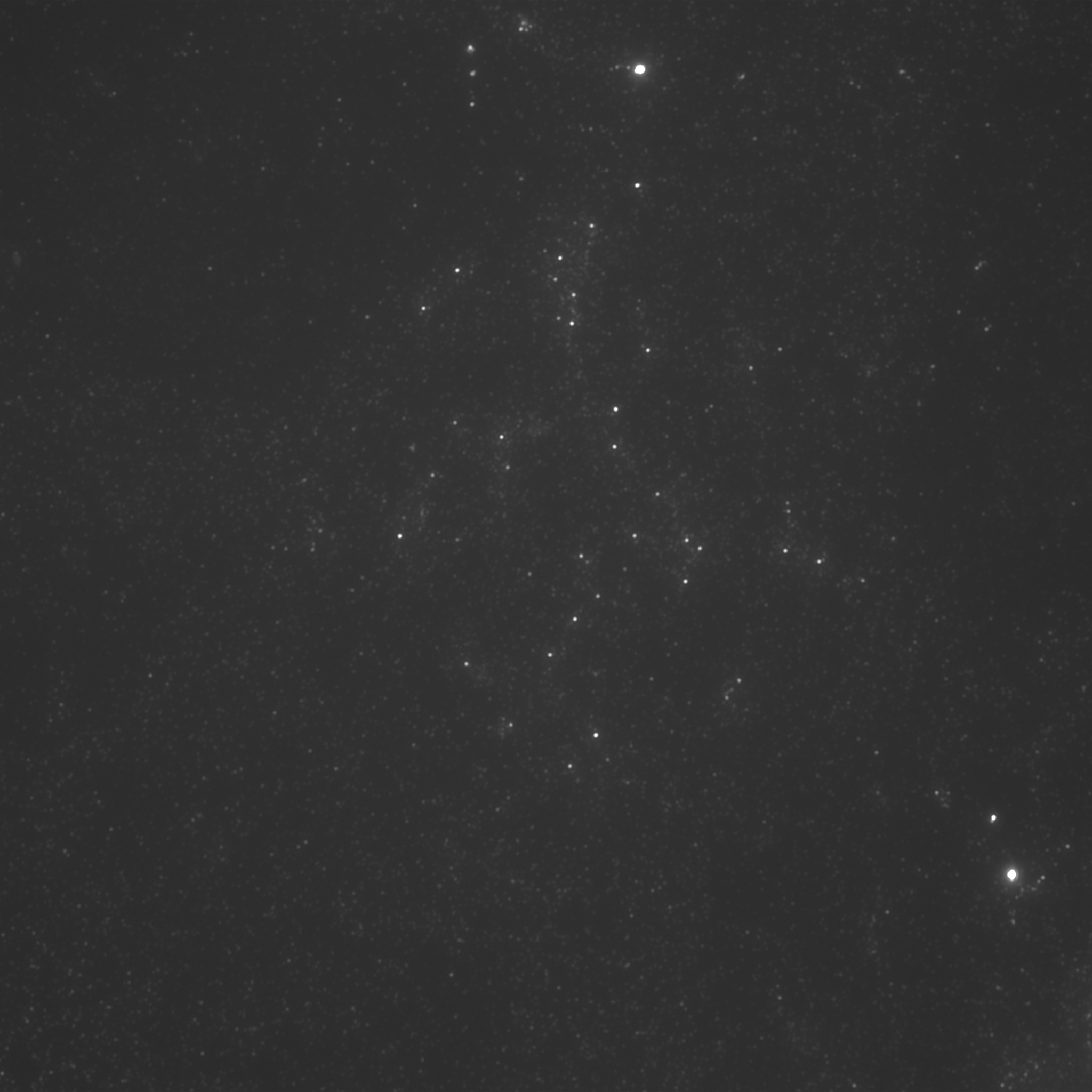

Supplement: Supplementary file 7 — Source data Fig. 4 [file 44319_2025_391_MOESM7_ESM.zip › Fig.4/B/KO/MLH1.tif]

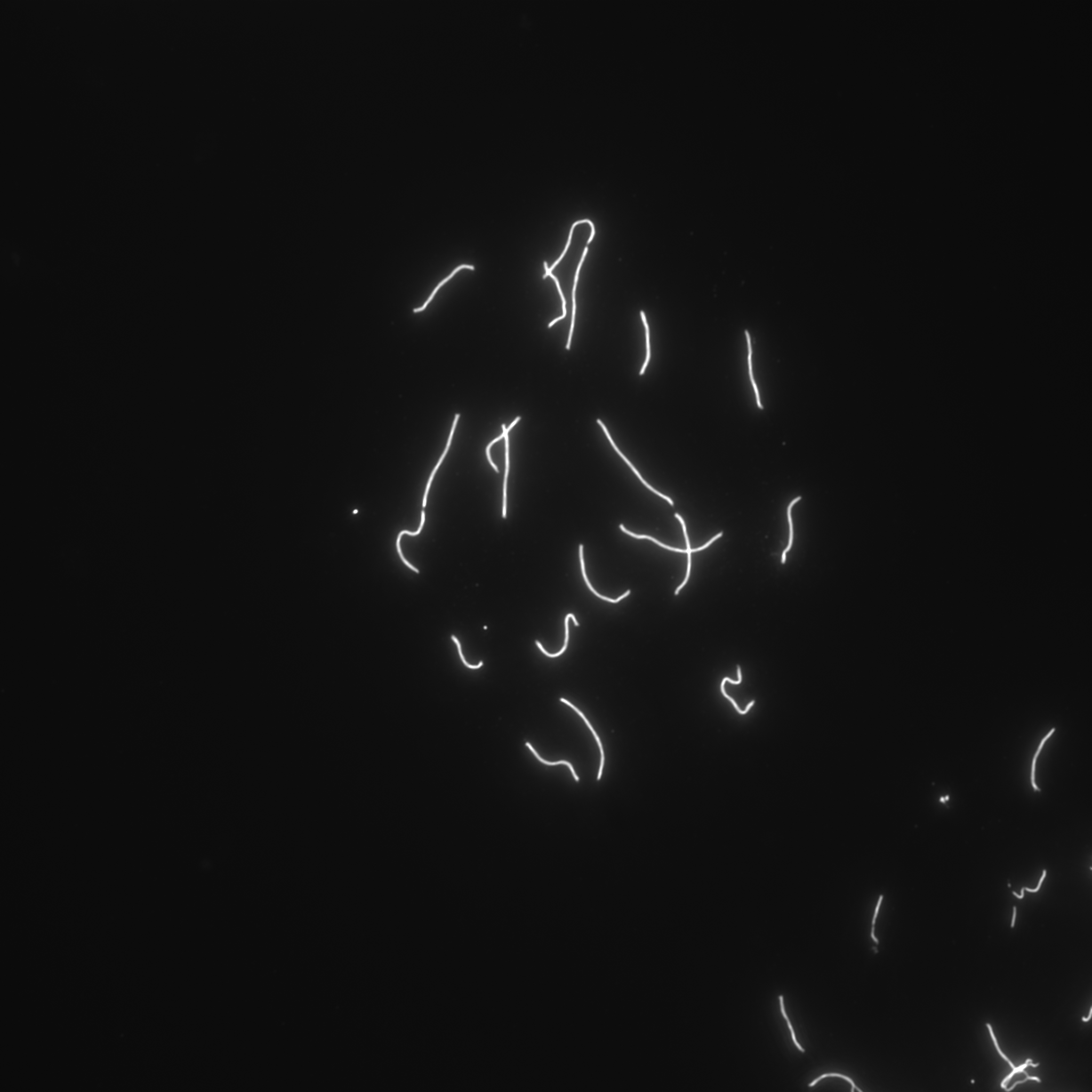

Supplement: Supplementary file 7 — Source data Fig. 4 [file 44319_2025_391_MOESM7_ESM.zip › Fig.4/B/KO/SYCP3.tif]

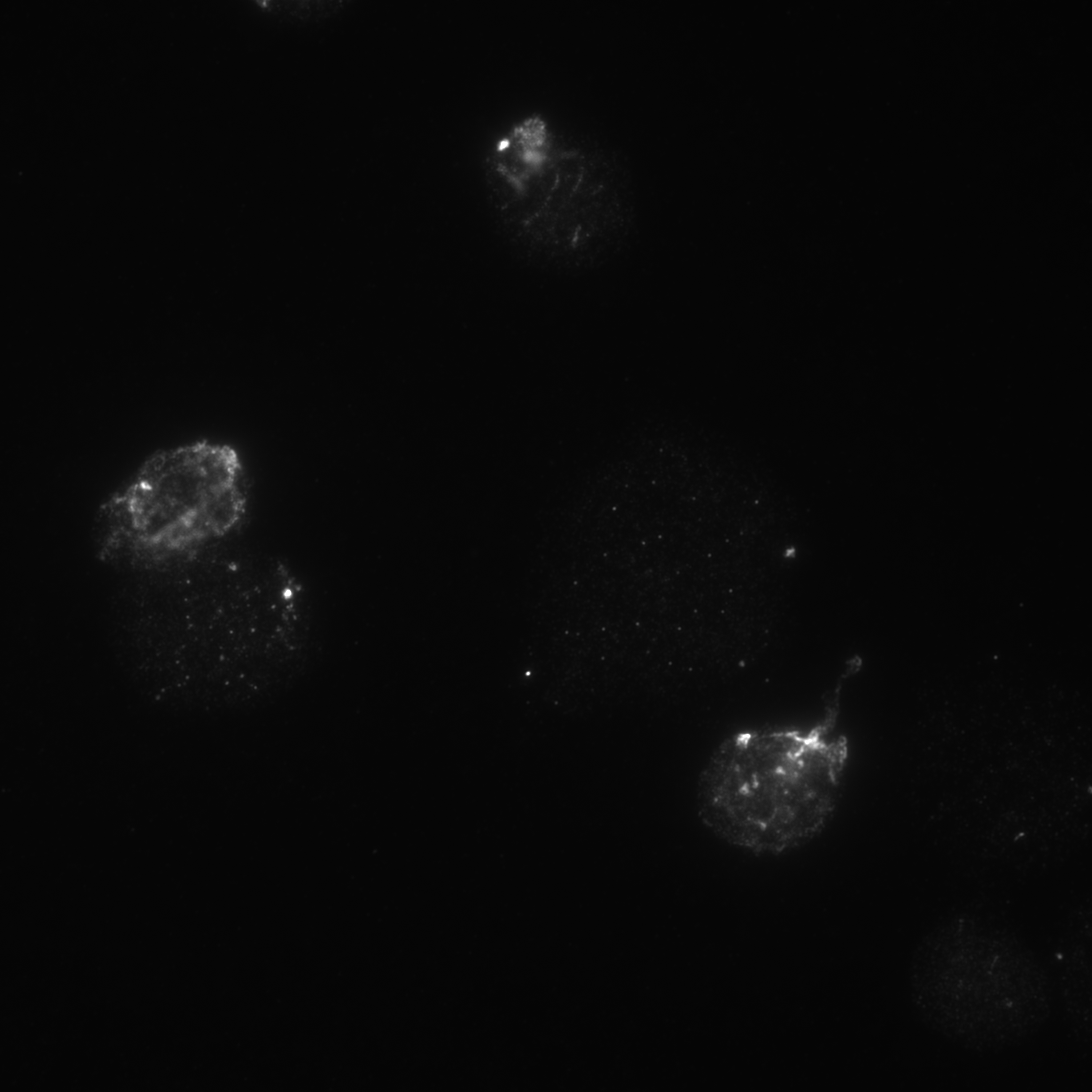

Supplement: Supplementary file 7 — Source data Fig. 4 [file 44319_2025_391_MOESM7_ESM.zip › Fig.4/B/WT/MLH1.tif]

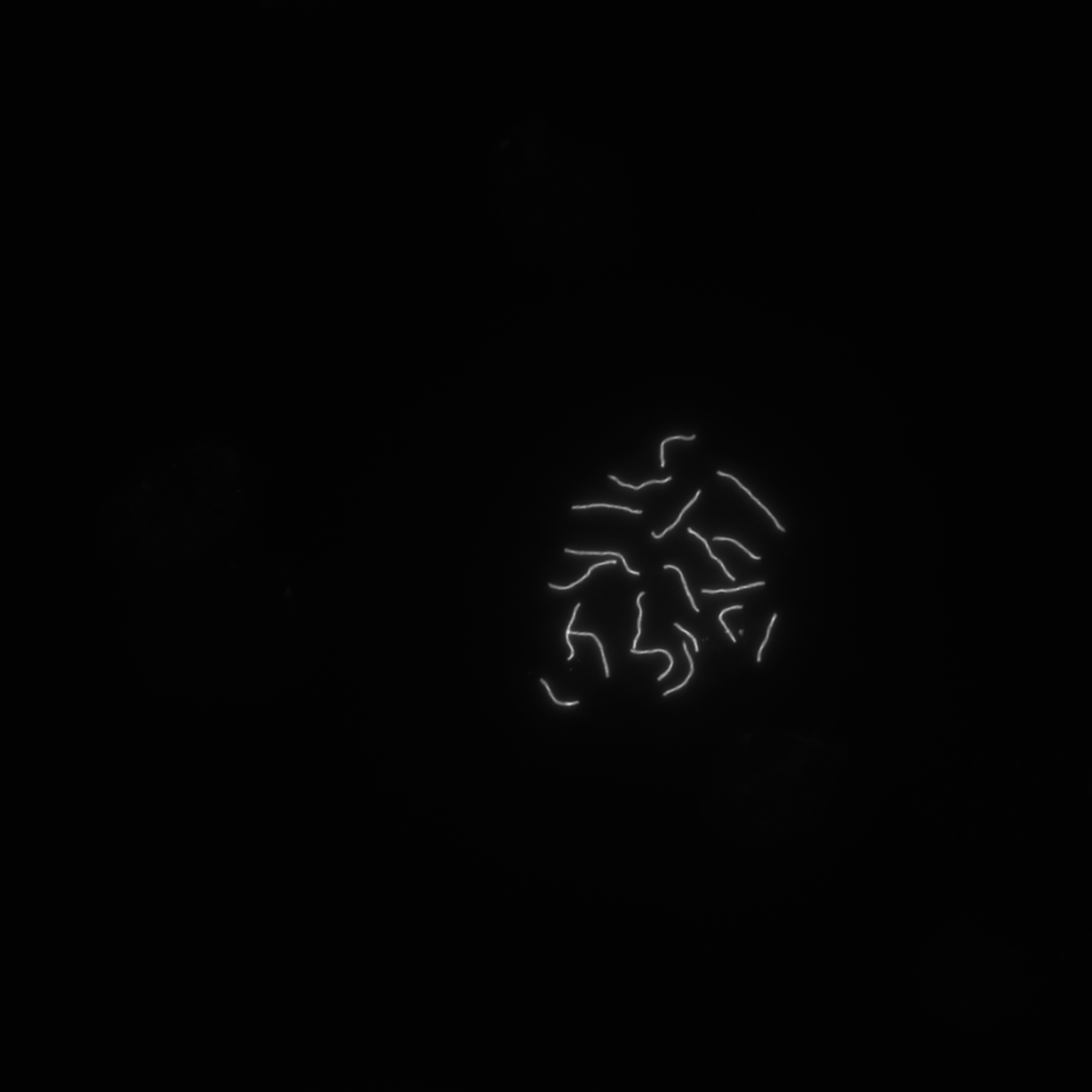

Supplement: Supplementary file 7 — Source data Fig. 4 [file 44319_2025_391_MOESM7_ESM.zip › Fig.4/B/WT/SYCP3.tif]

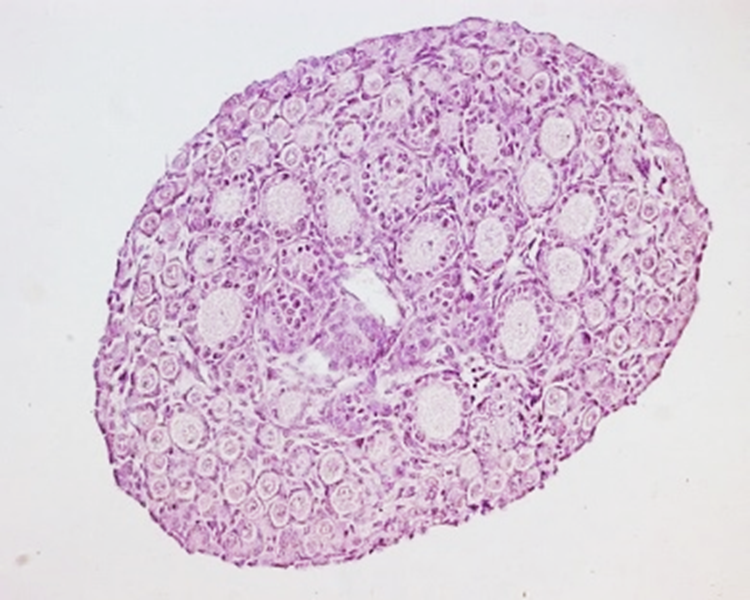

Supplement: Supplementary file 7 — Source data Fig. 4 [file 44319_2025_391_MOESM7_ESM.zip › Fig.4/E/Control/Control.png]

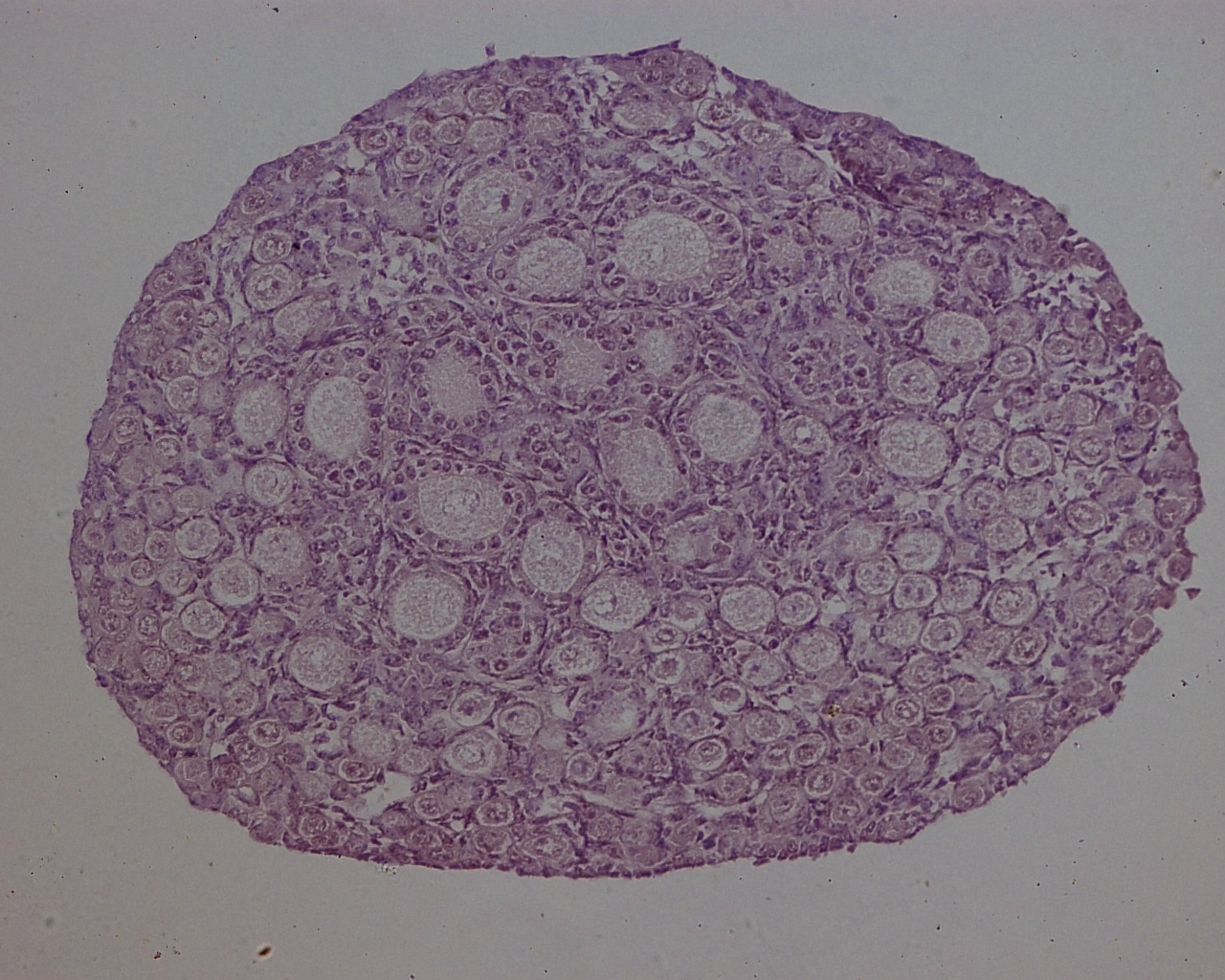

Supplement: Supplementary file 7 — Source data Fig. 4 [file 44319_2025_391_MOESM7_ESM.zip › Fig.4/E/KO/KO-20X.tif]

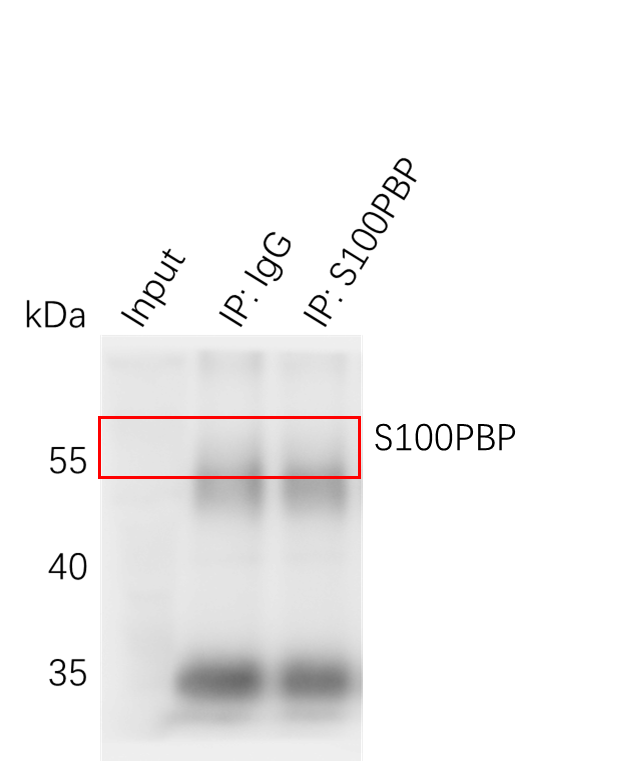

Supplement: Supplementary file 8 — Source data Fig. 5 [file 44319_2025_391_MOESM8_ESM.zip › Fig.5/B/S100pbp-KO-S100PBP.png]

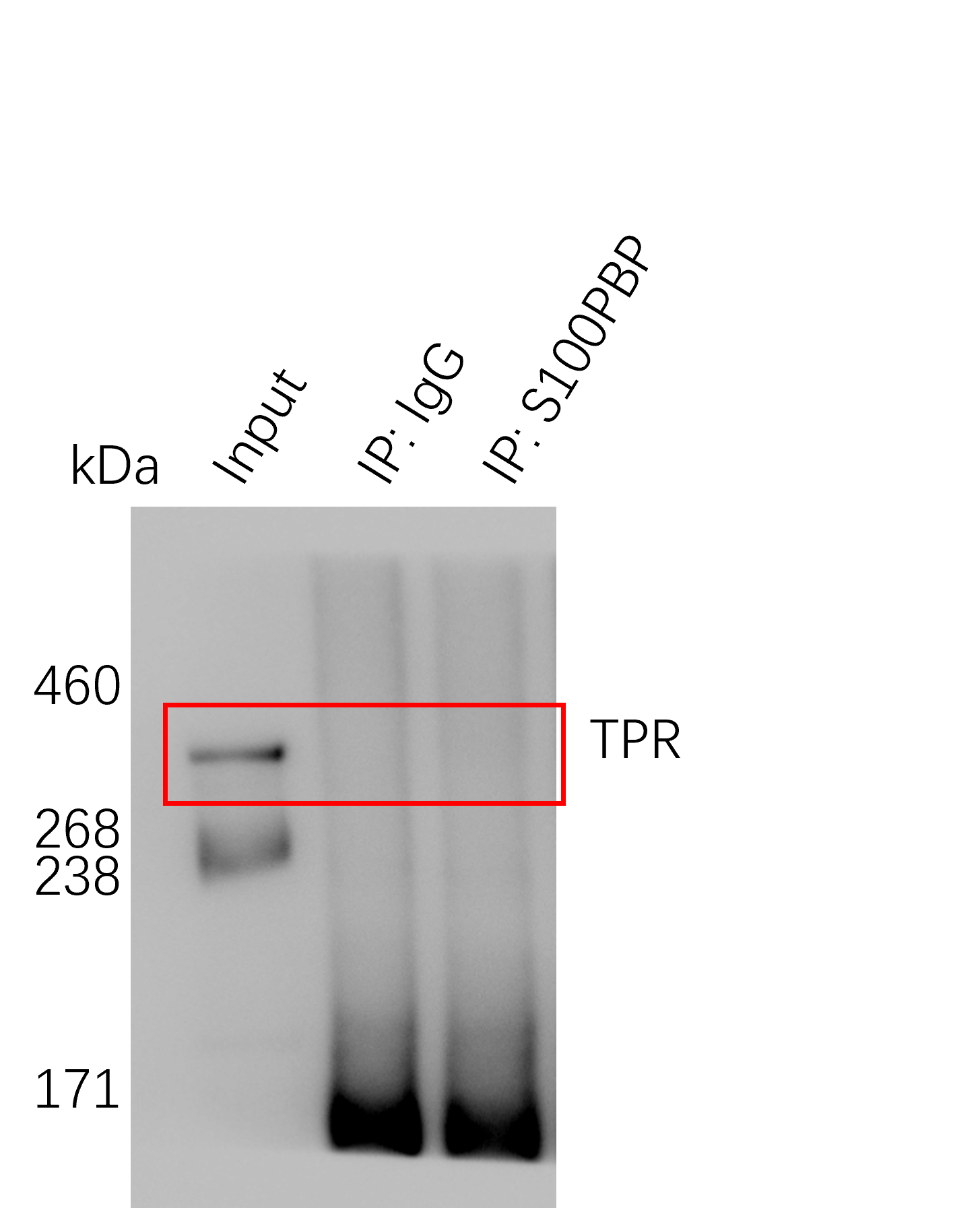

Supplement: Supplementary file 8 — Source data Fig. 5 [file 44319_2025_391_MOESM8_ESM.zip › Fig.5/B/S100PBP-KO-TPR.png]

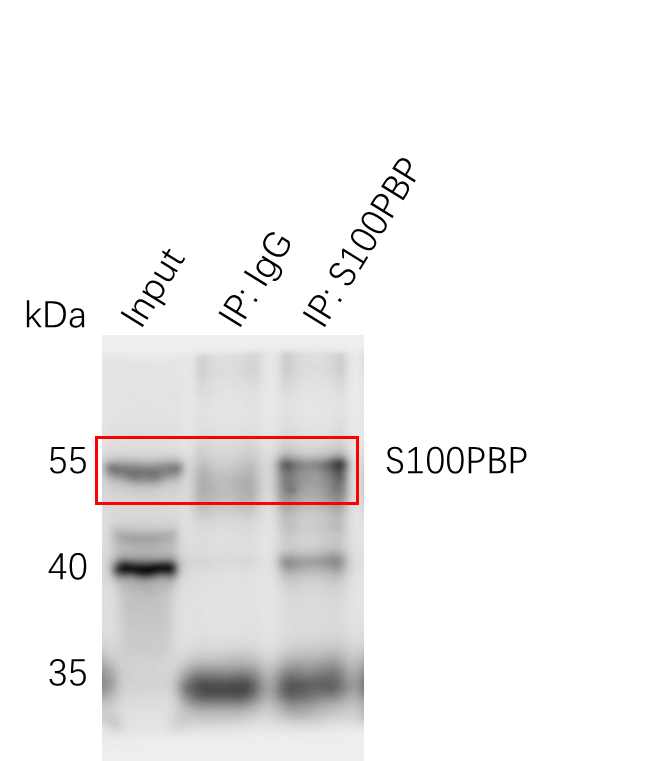

Supplement: Supplementary file 8 — Source data Fig. 5 [file 44319_2025_391_MOESM8_ESM.zip › Fig.5/B/WT-S100PBP.png]

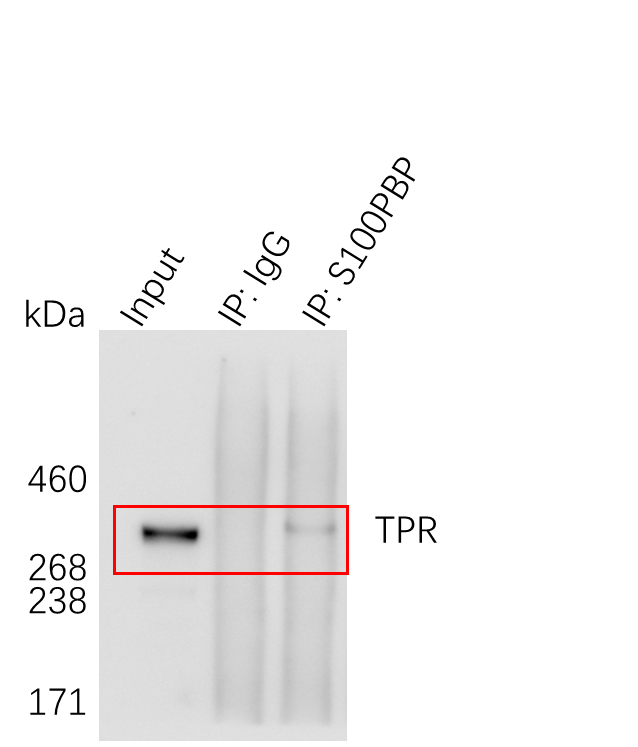

Supplement: Supplementary file 8 — Source data Fig. 5 [file 44319_2025_391_MOESM8_ESM.zip › Fig.5/B/WT-TPR.png]

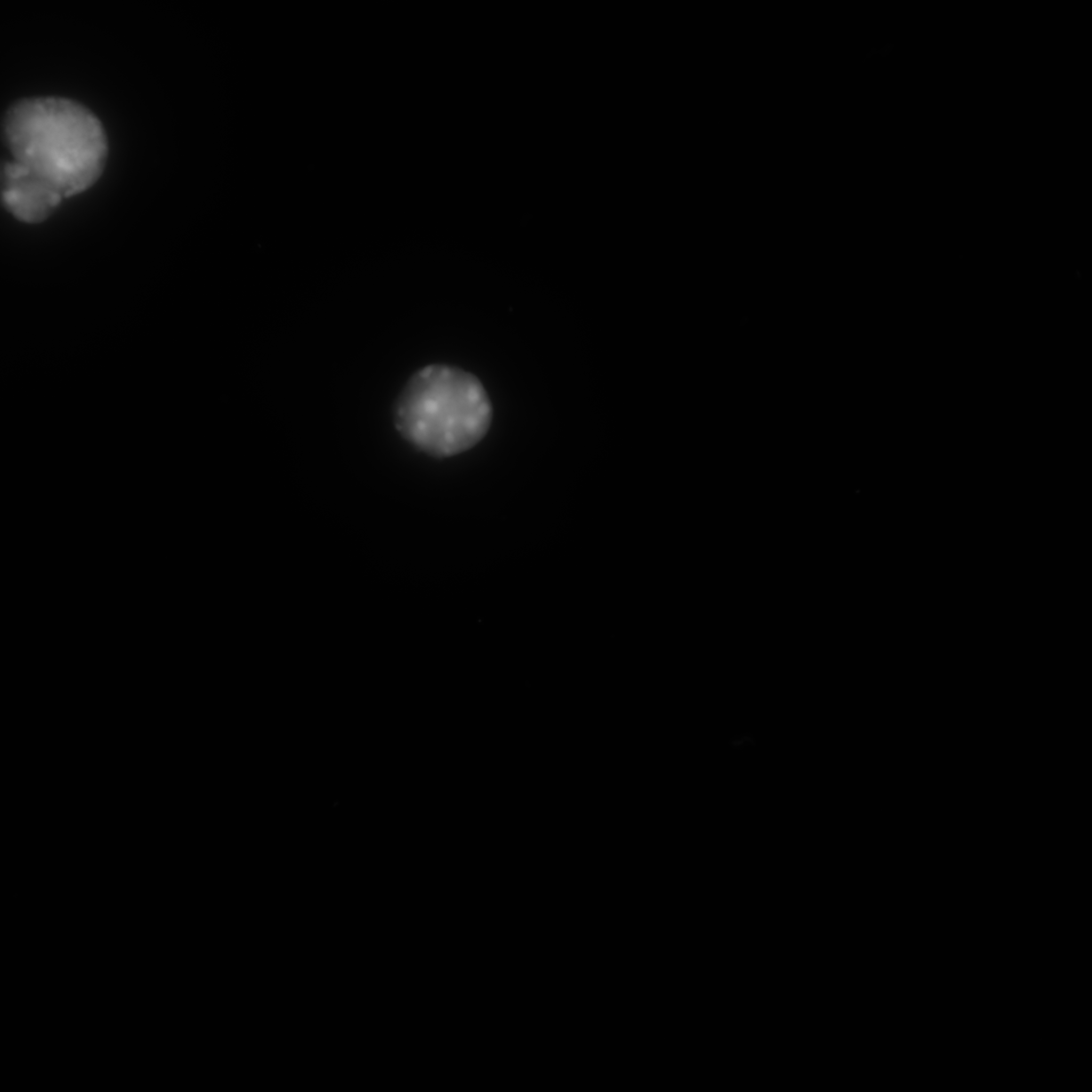

Supplement: Supplementary file 8 — Source data Fig. 5 [file 44319_2025_391_MOESM8_ESM.zip › Fig.5/E/Control/Hoechst.tif]

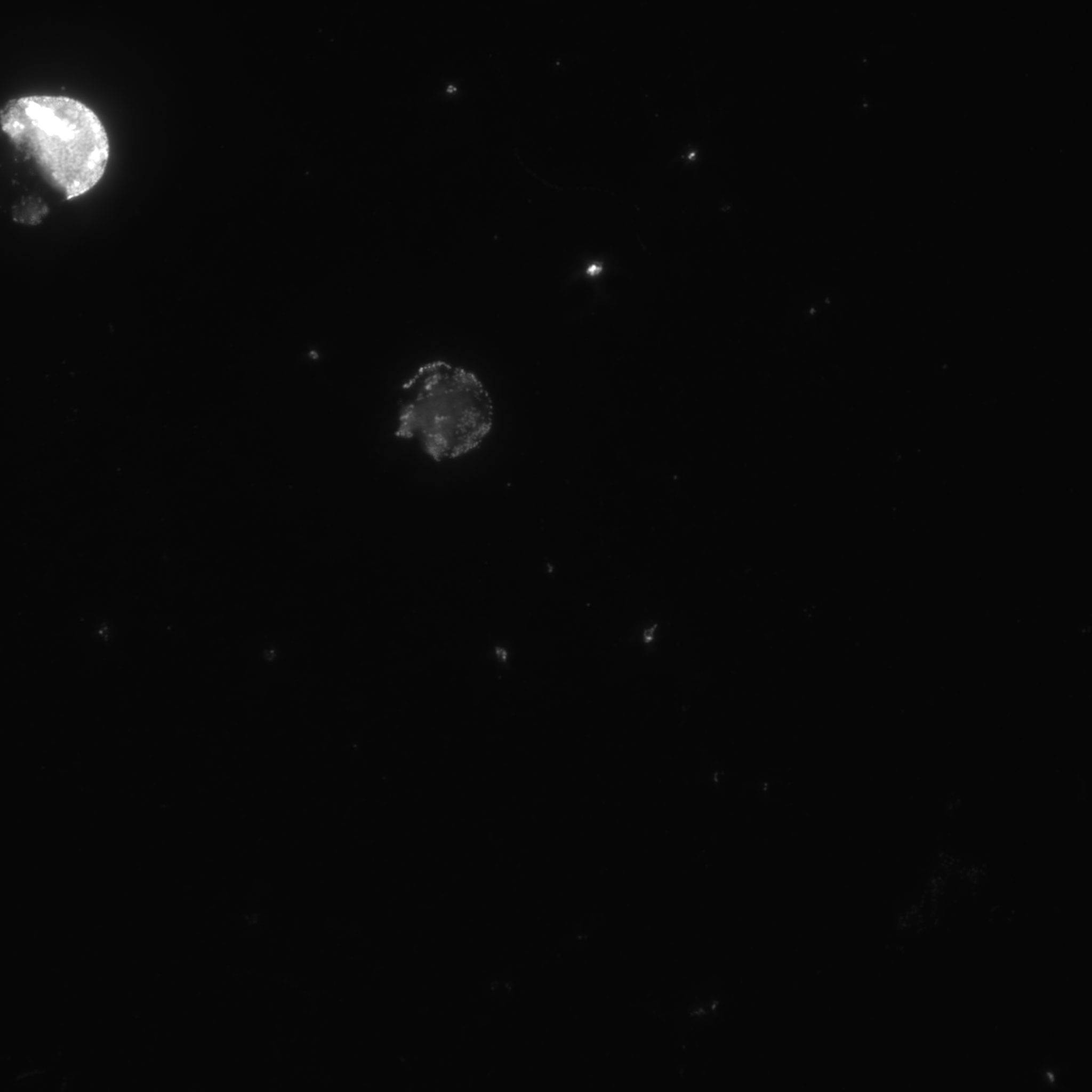

Supplement: Supplementary file 8 — Source data Fig. 5 [file 44319_2025_391_MOESM8_ESM.zip › Fig.5/E/Control/S100PBP.tif]

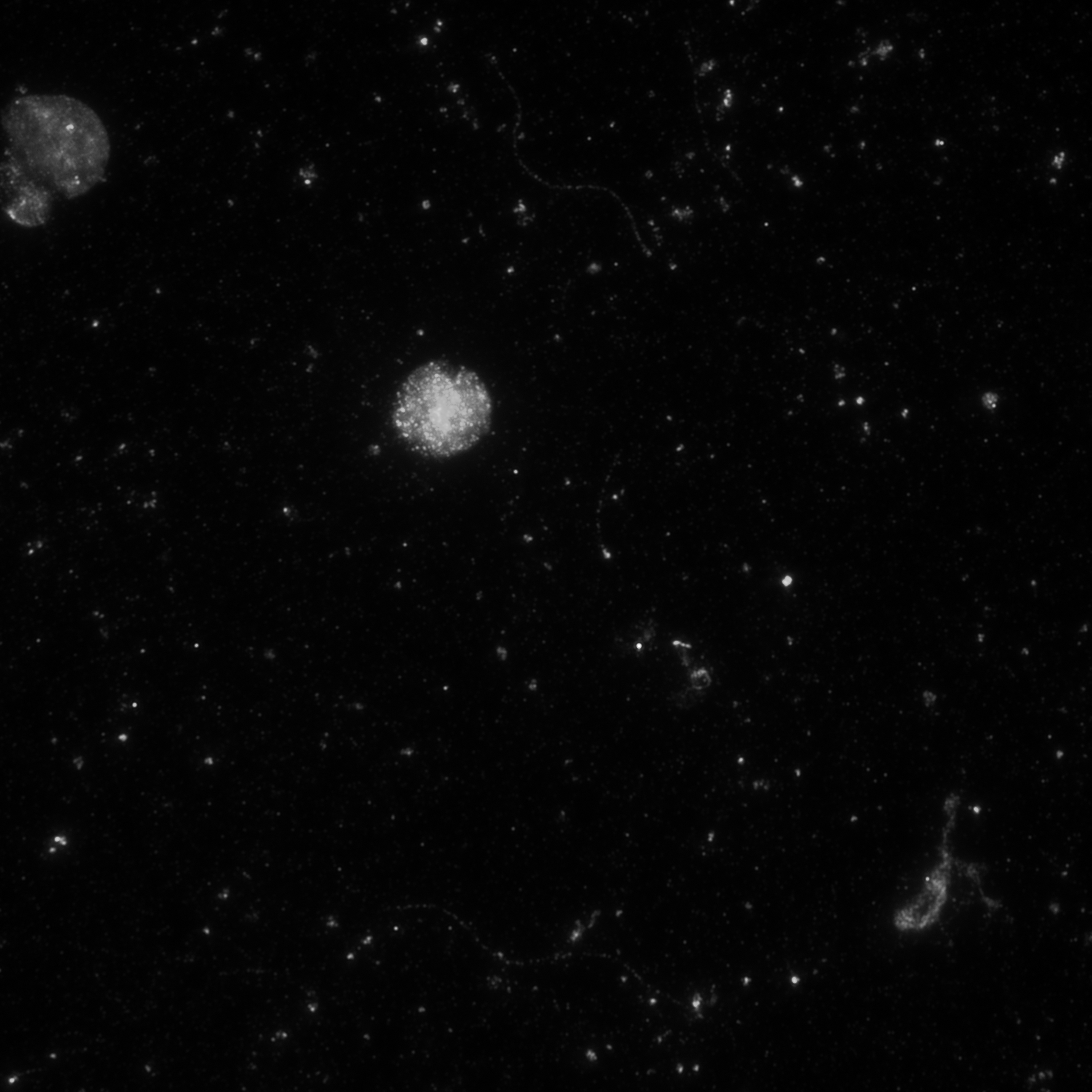

Supplement: Supplementary file 8 — Source data Fig. 5 [file 44319_2025_391_MOESM8_ESM.zip › Fig.5/E/Control/SYCP3.tif]

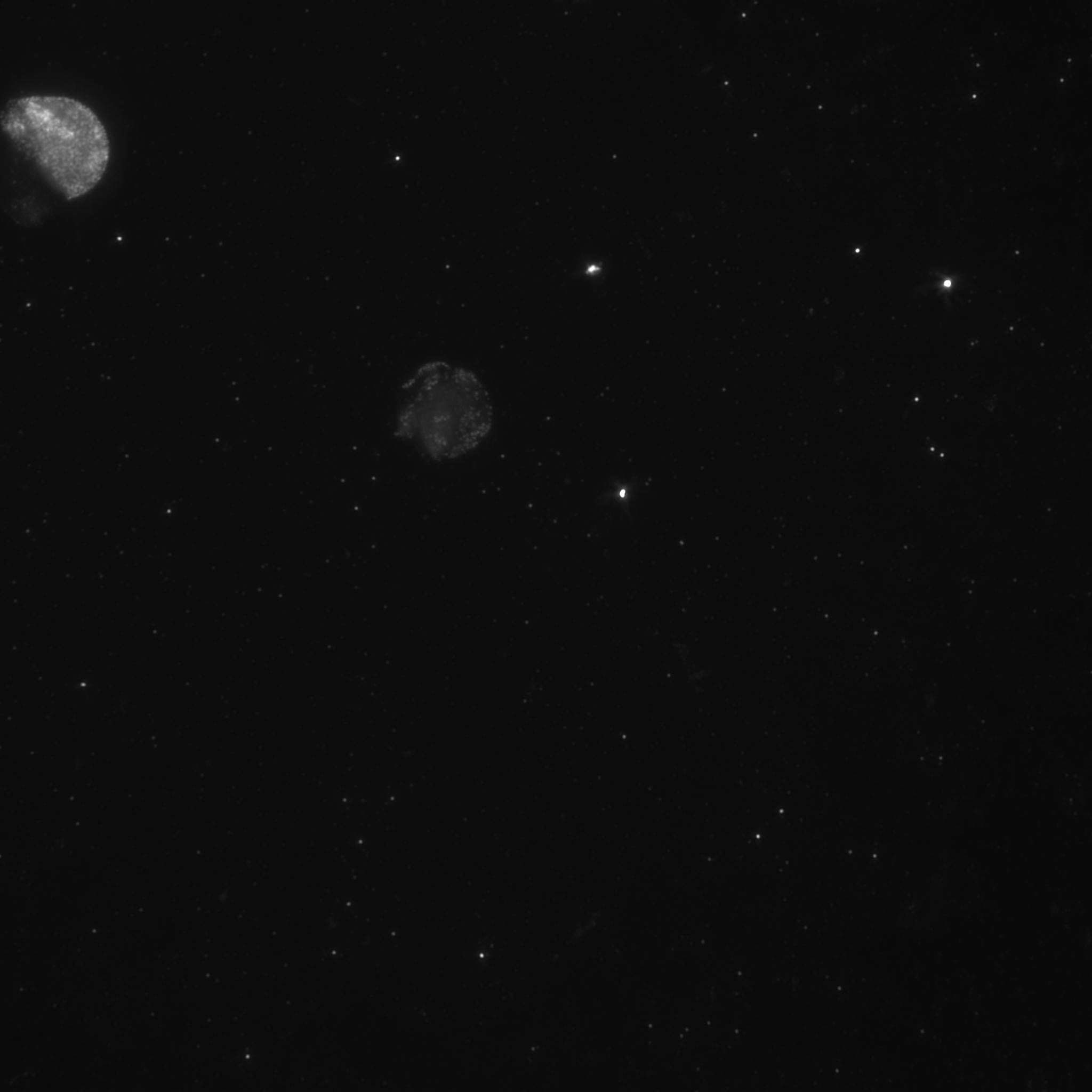

Supplement: Supplementary file 8 — Source data Fig. 5 [file 44319_2025_391_MOESM8_ESM.zip › Fig.5/E/Control/TPR.tif]

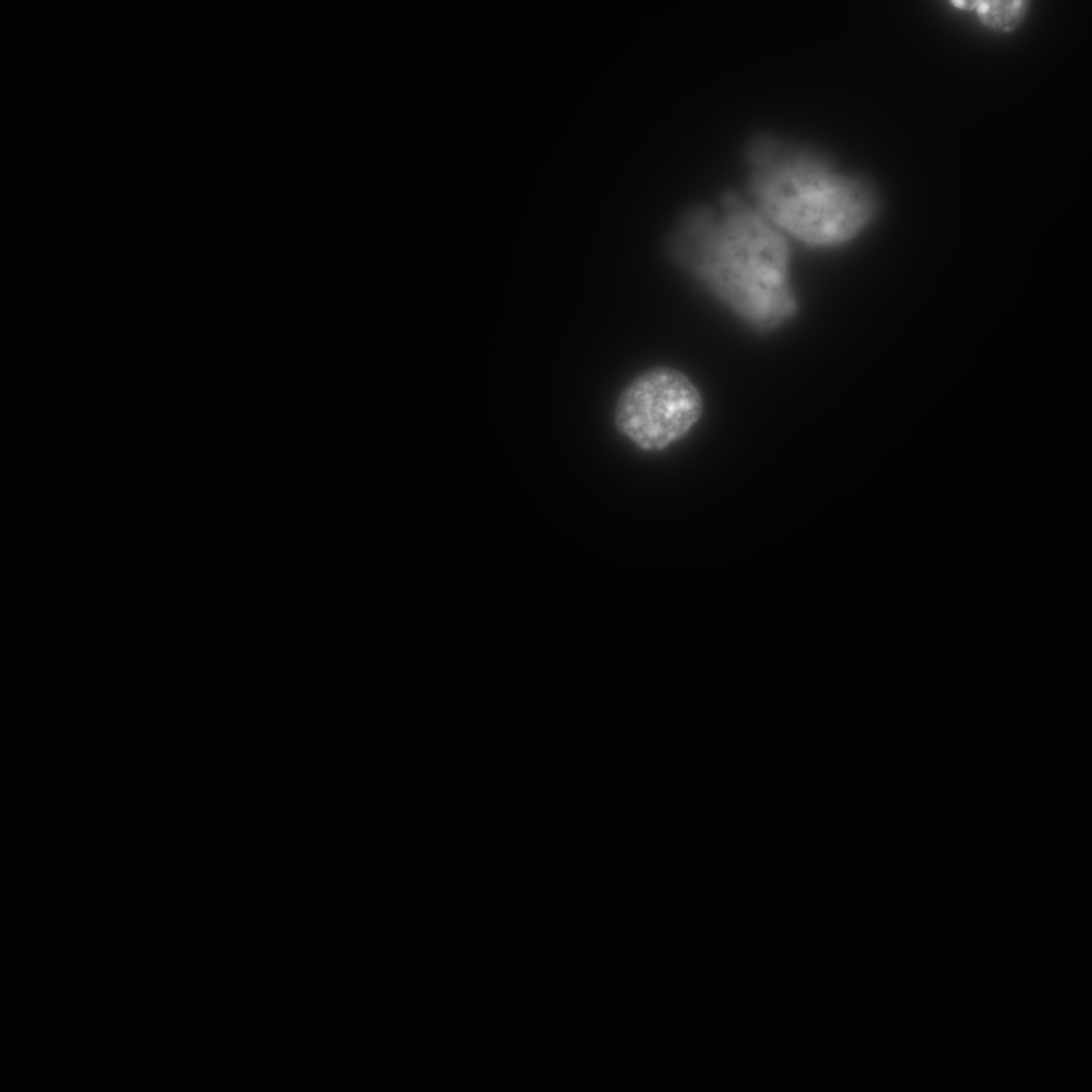

Supplement: Supplementary file 8 — Source data Fig. 5 [file 44319_2025_391_MOESM8_ESM.zip › Fig.5/E/Stra8,Tpr-cKO/Hoechst.tif]

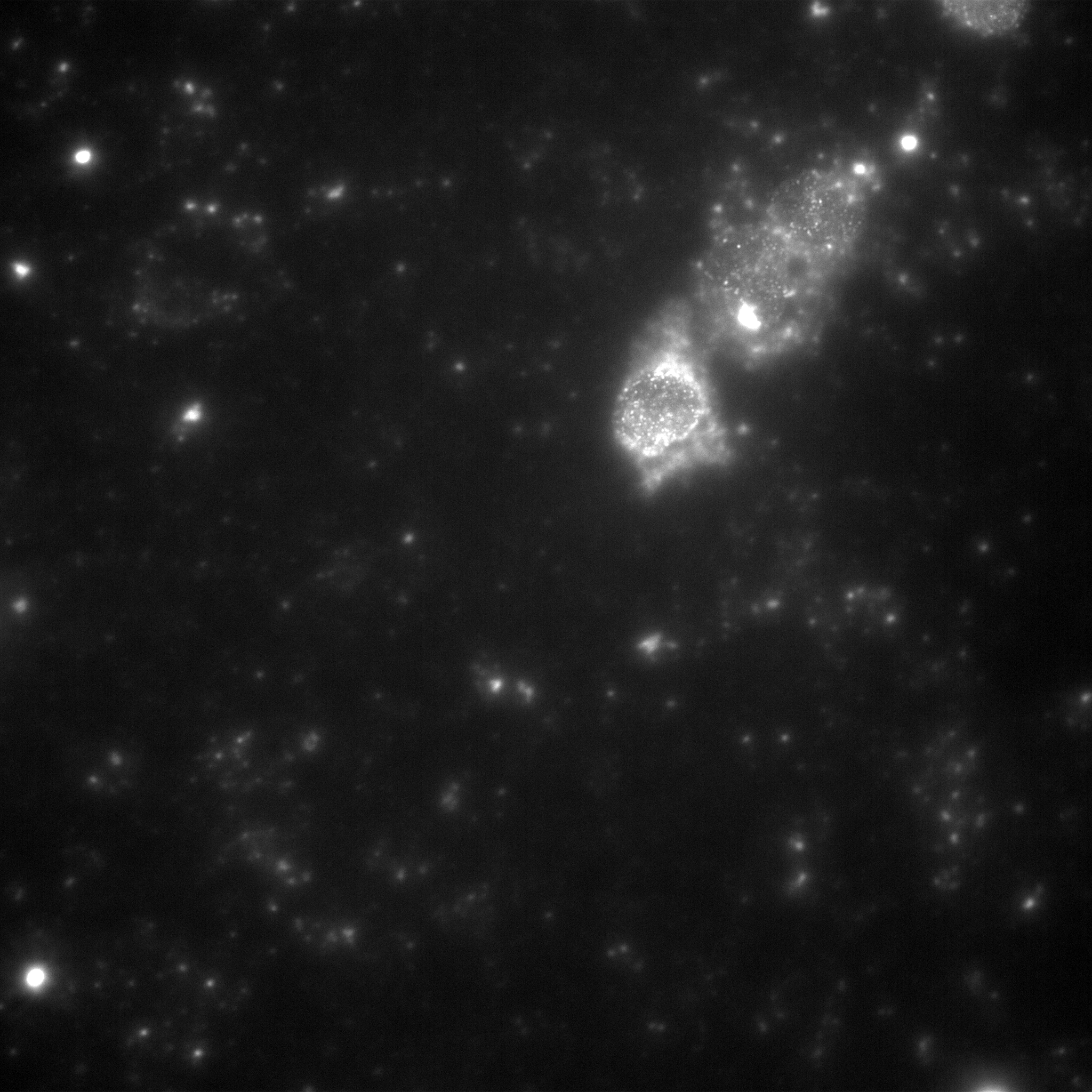

Supplement: Supplementary file 8 — Source data Fig. 5 [file 44319_2025_391_MOESM8_ESM.zip › Fig.5/E/Stra8,Tpr-cKO/S100PBP.tif]

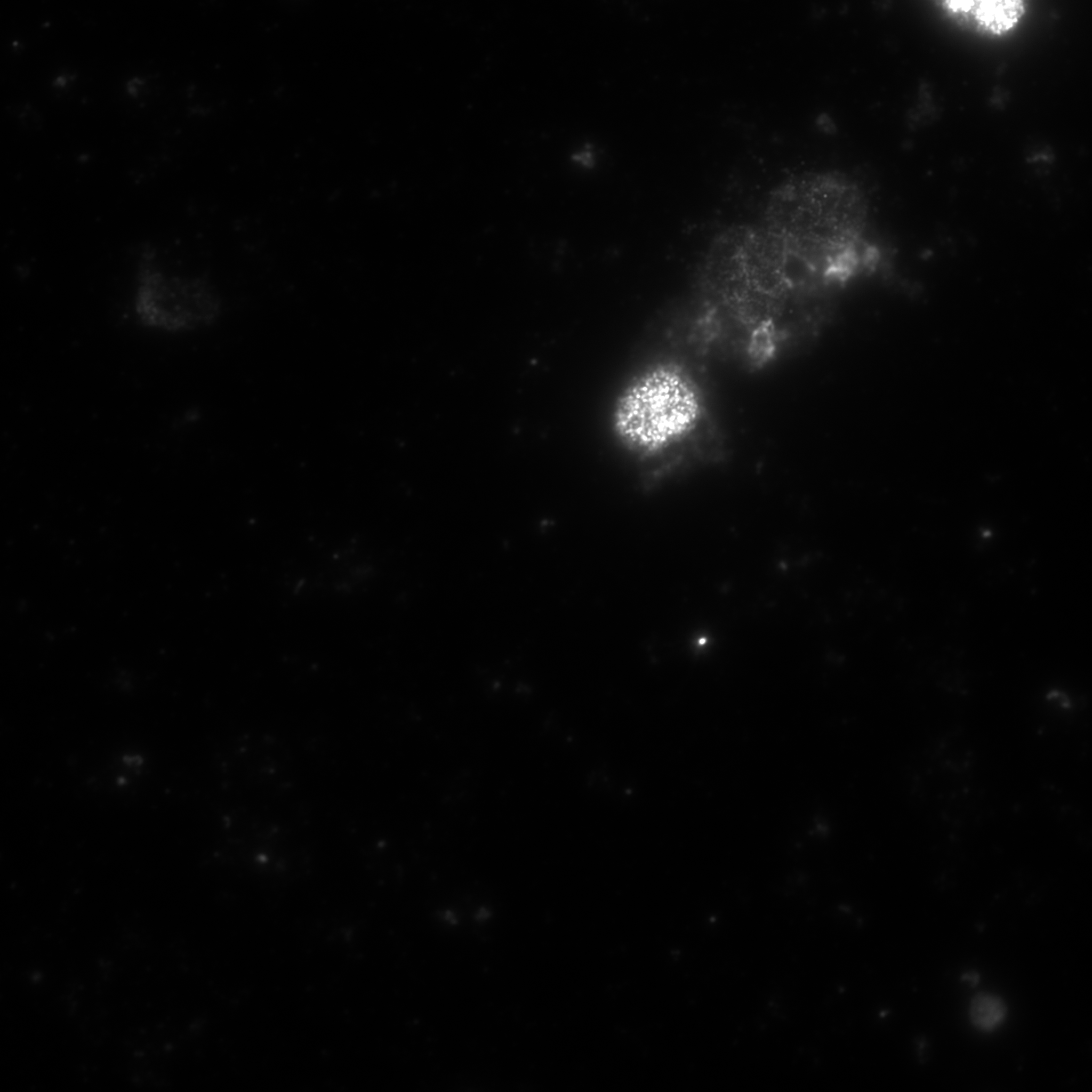

Supplement: Supplementary file 8 — Source data Fig. 5 [file 44319_2025_391_MOESM8_ESM.zip › Fig.5/E/Stra8,Tpr-cKO/SYCP2.tif]

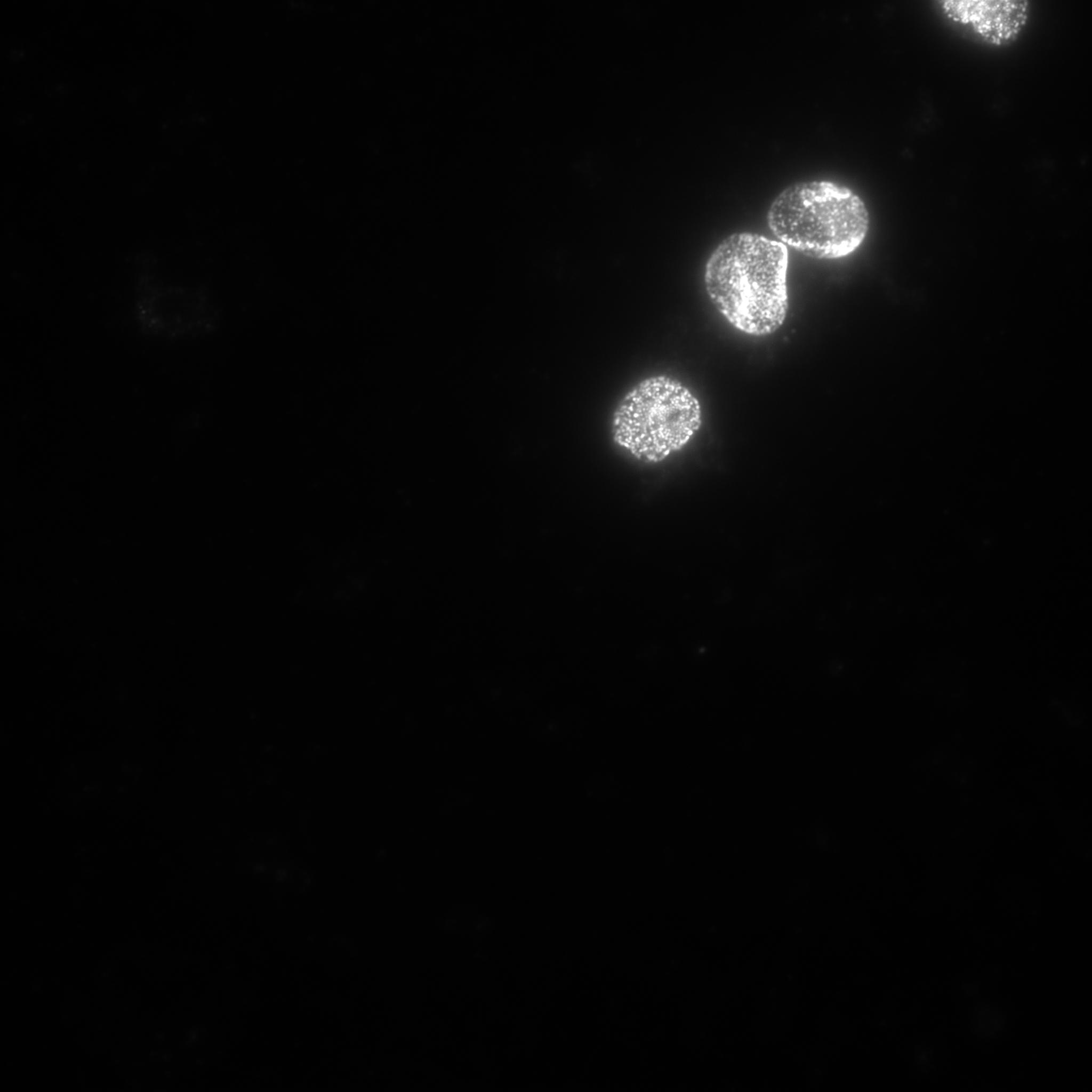

Supplement: Supplementary file 8 — Source data Fig. 5 [file 44319_2025_391_MOESM8_ESM.zip › Fig.5/E/Stra8,Tpr-cKO/TPR.tif]

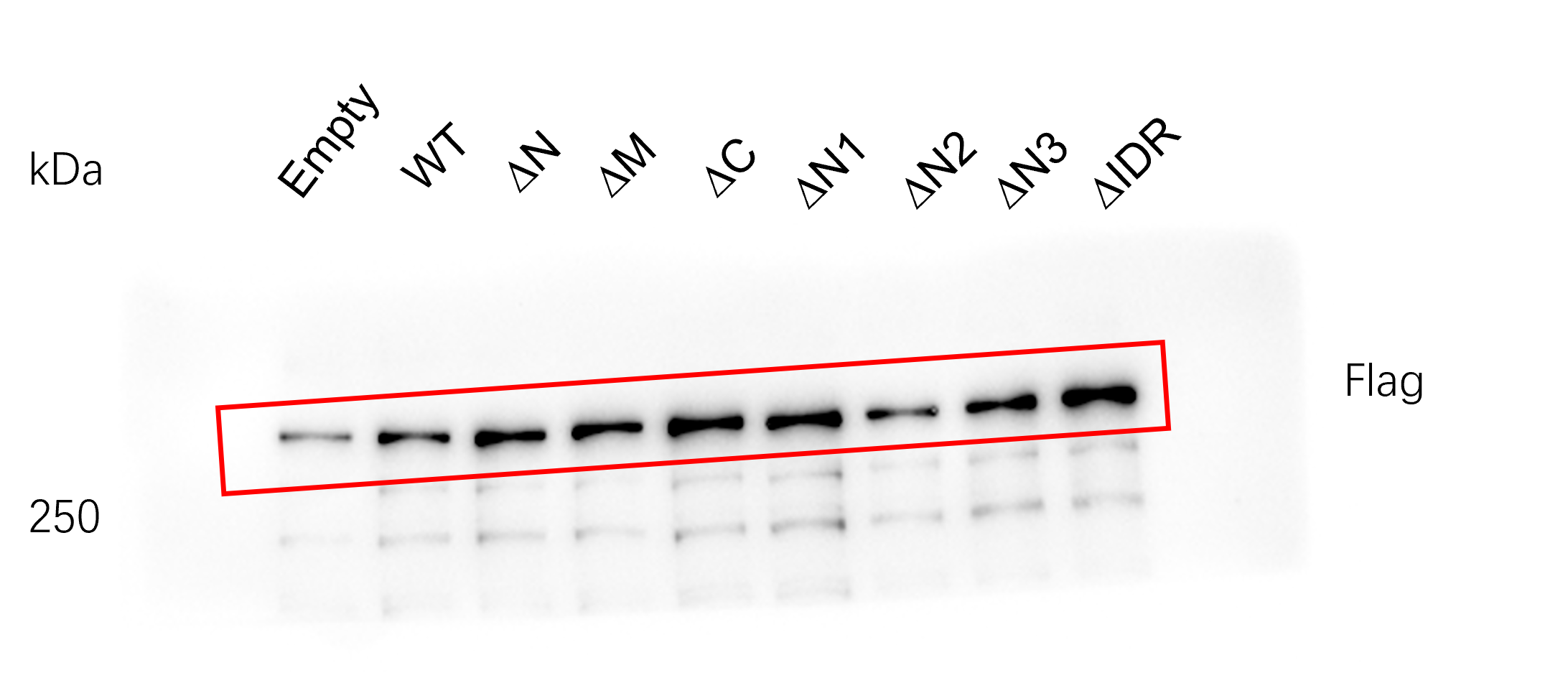

Supplement: Supplementary file 9 — Source data Fig. 6 [file 44319_2025_391_MOESM9_ESM.zip › Fig.6/B/Input-Flag.png]

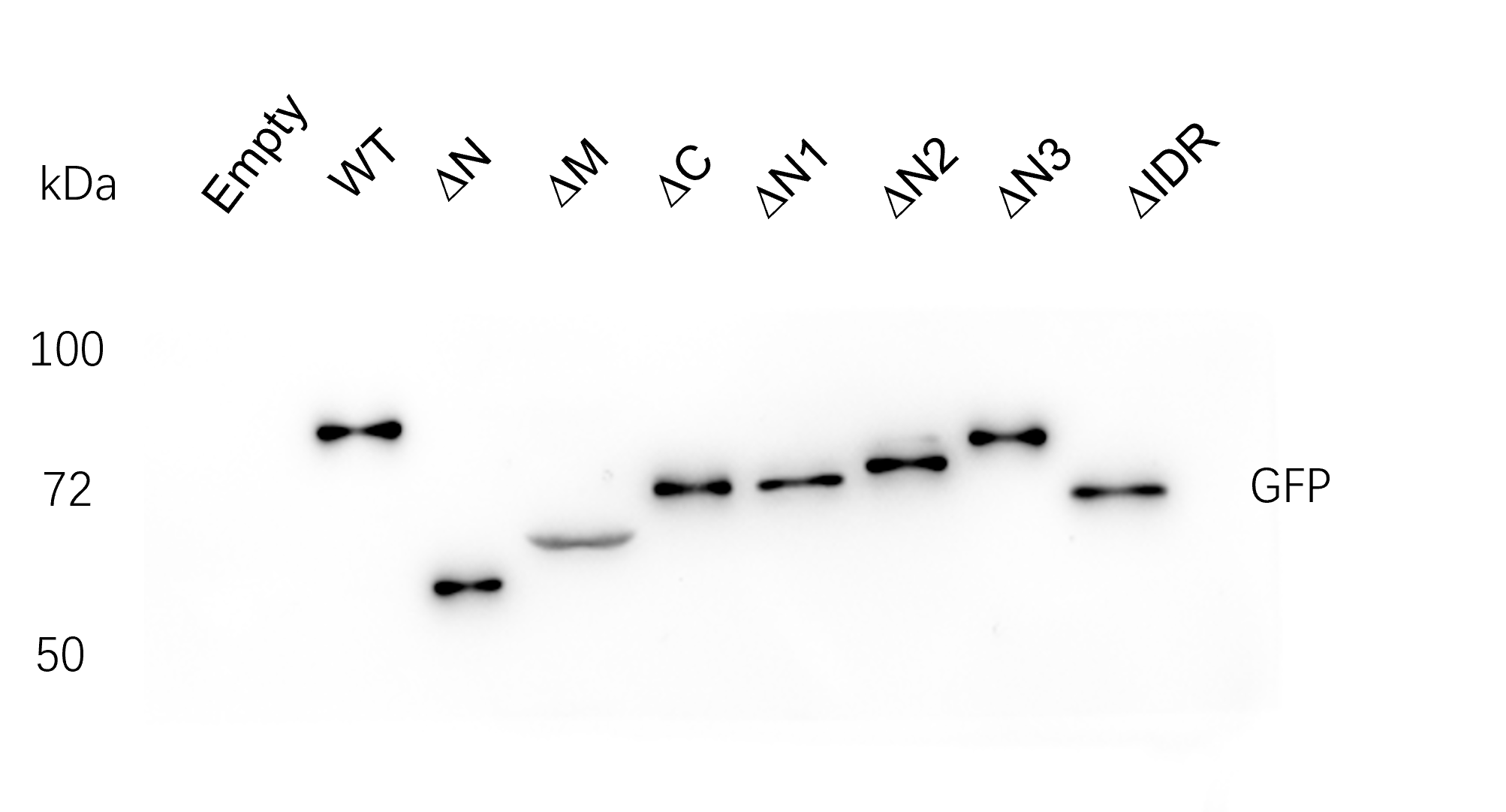

Supplement: Supplementary file 9 — Source data Fig. 6 [file 44319_2025_391_MOESM9_ESM.zip › Fig.6/B/Input-GFP.png]

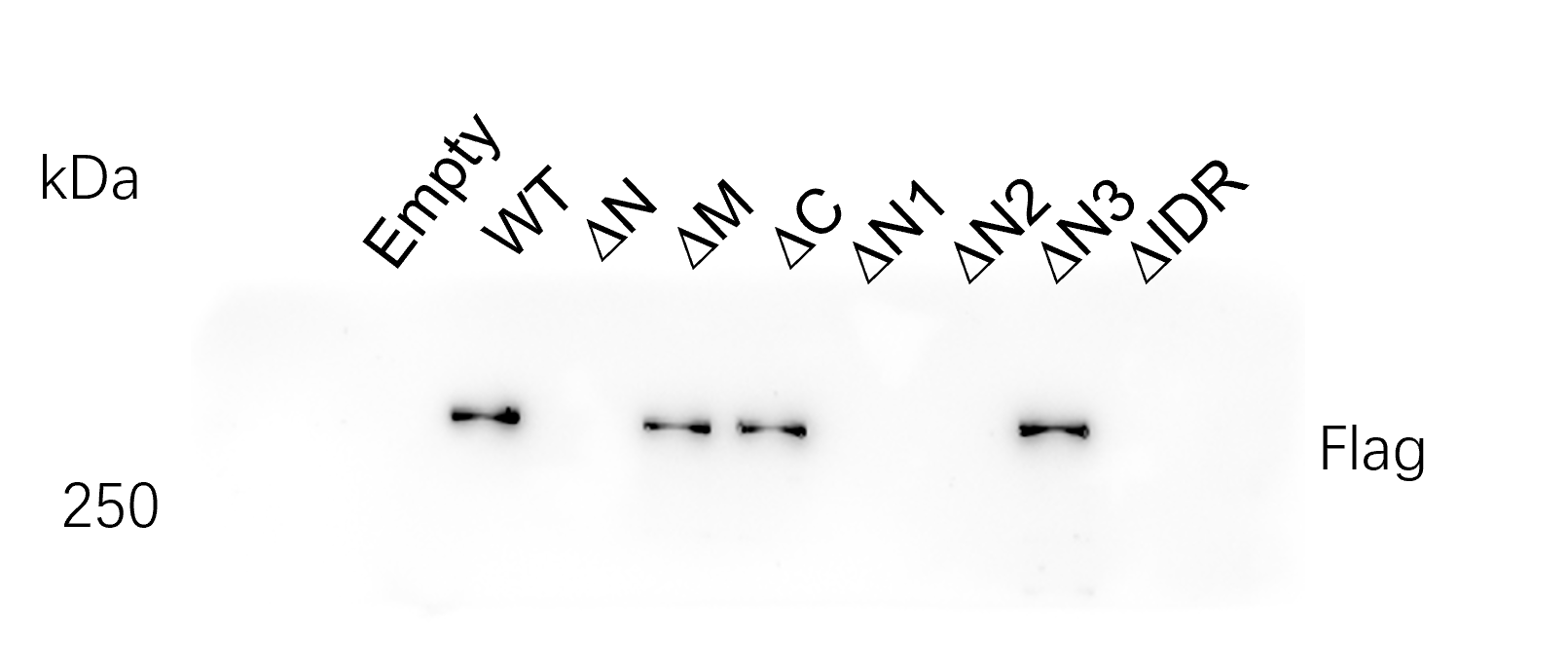

Supplement: Supplementary file 9 — Source data Fig. 6 [file 44319_2025_391_MOESM9_ESM.zip › Fig.6/B/IP-Flag.png]

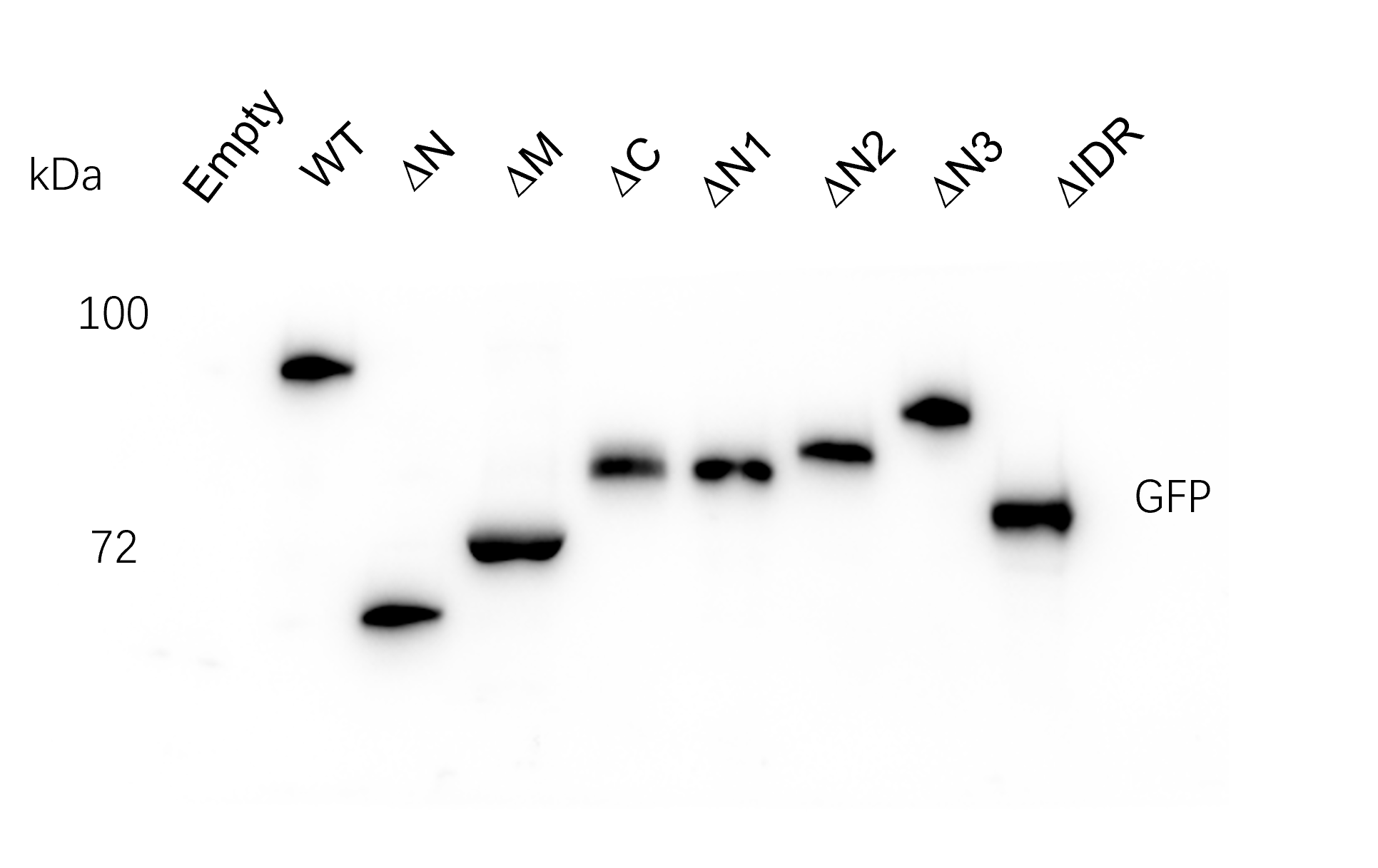

Supplement: Supplementary file 9 — Source data Fig. 6 [file 44319_2025_391_MOESM9_ESM.zip › Fig.6/B/IP-GFP.png]
