# Supplementary material for: S100PBP interacts with nucleoporin TPR and facilitates XY crossover formation in mice
Source: EMBO Rep. 2025 Apr 9;26(9):2280–99. doi: 10.1038/s44319-025-00391-y (PMC12069632; doi:10.1038/s44319-025-00391-y)
Supplement: Supplementary file 10 — Expanded View Figures [file 44319_2025_391_MOESM10_ESM.pdf]

Expanded View Figures

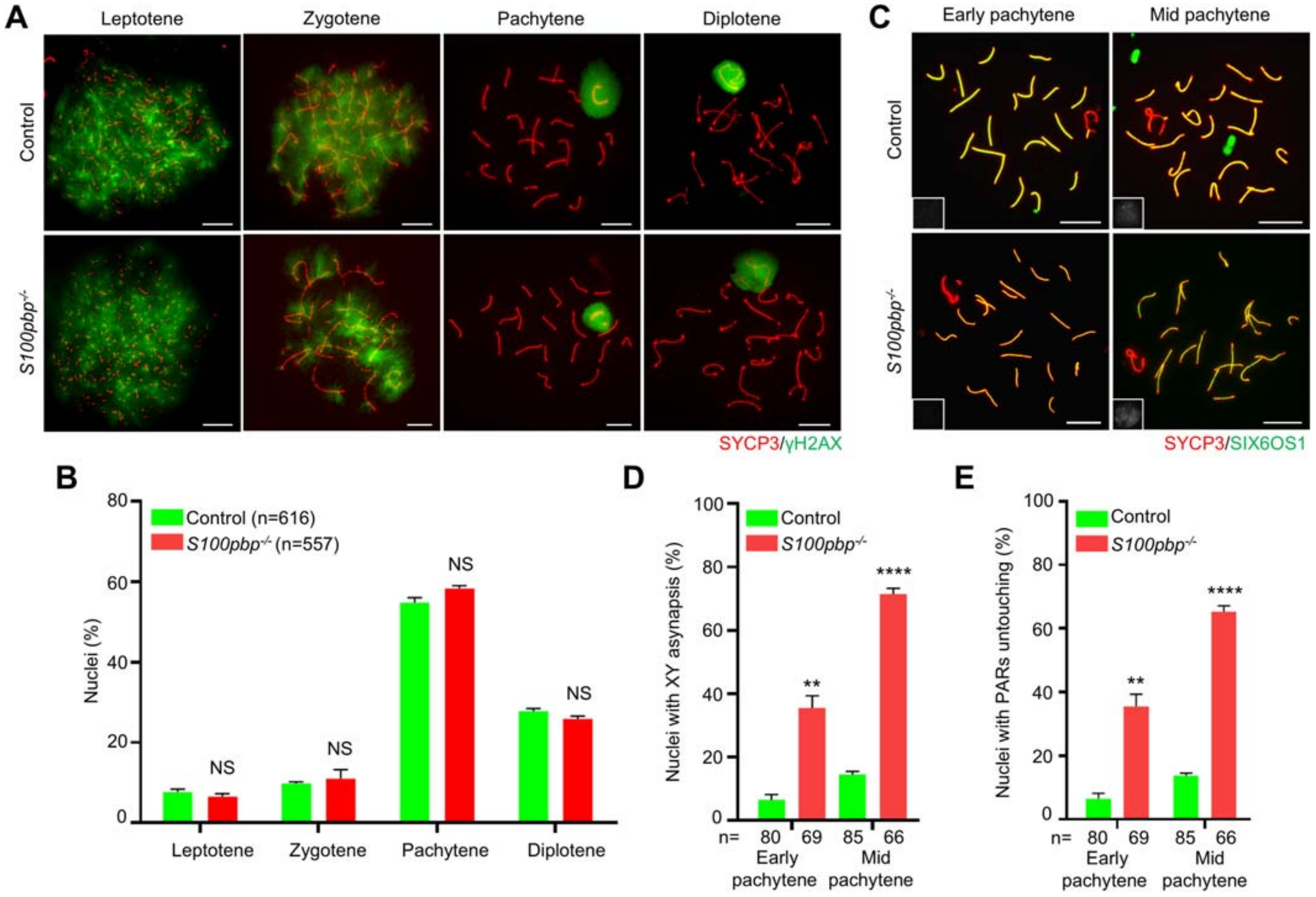

Figure EV1. The progression of meiotic prophase I analysis of *S100pbp*$^{-/-}$ mice.

(**A**) Immunofluorescence staining of SYCP3 (red) and γH2AX (green) on spermatocyte spreads from 8-week-old control and *S100pbp*$^{-/-}$ mice. Scale bars, 10 μm. (**B**) The percentages of spermatocytes of each substage of meiotic prophase I in 8-week-old control and *S100pbp*$^{-/-}$ mice. Data represent the mean ± SEM from at least three biological replicates. *n*, the number of cells scored. NS, not significant; two-tailed Student's *t* test. (**C**) Representative spread spermatocytes stained for the lateral element (SYCP3, red) and the central element (SIX6OS1, green) at the early, mid or late pachytene stages. Miniaturized H1t staining (gray), shown in the lower-left corner of the overlay images. Scale bars, 10 μm. (**D**, **E**) Frequencies of nuclei with XY asynapsis (**D**) and nuclei with XY PARs untouching (**E**). Data represent the mean ± SEM from at least three biological replicates. *n*, the number of cells scored. ***P* = 0.0022; *****P* < 0.0001; two-tailed Student's *t* test.

© The Author(s)

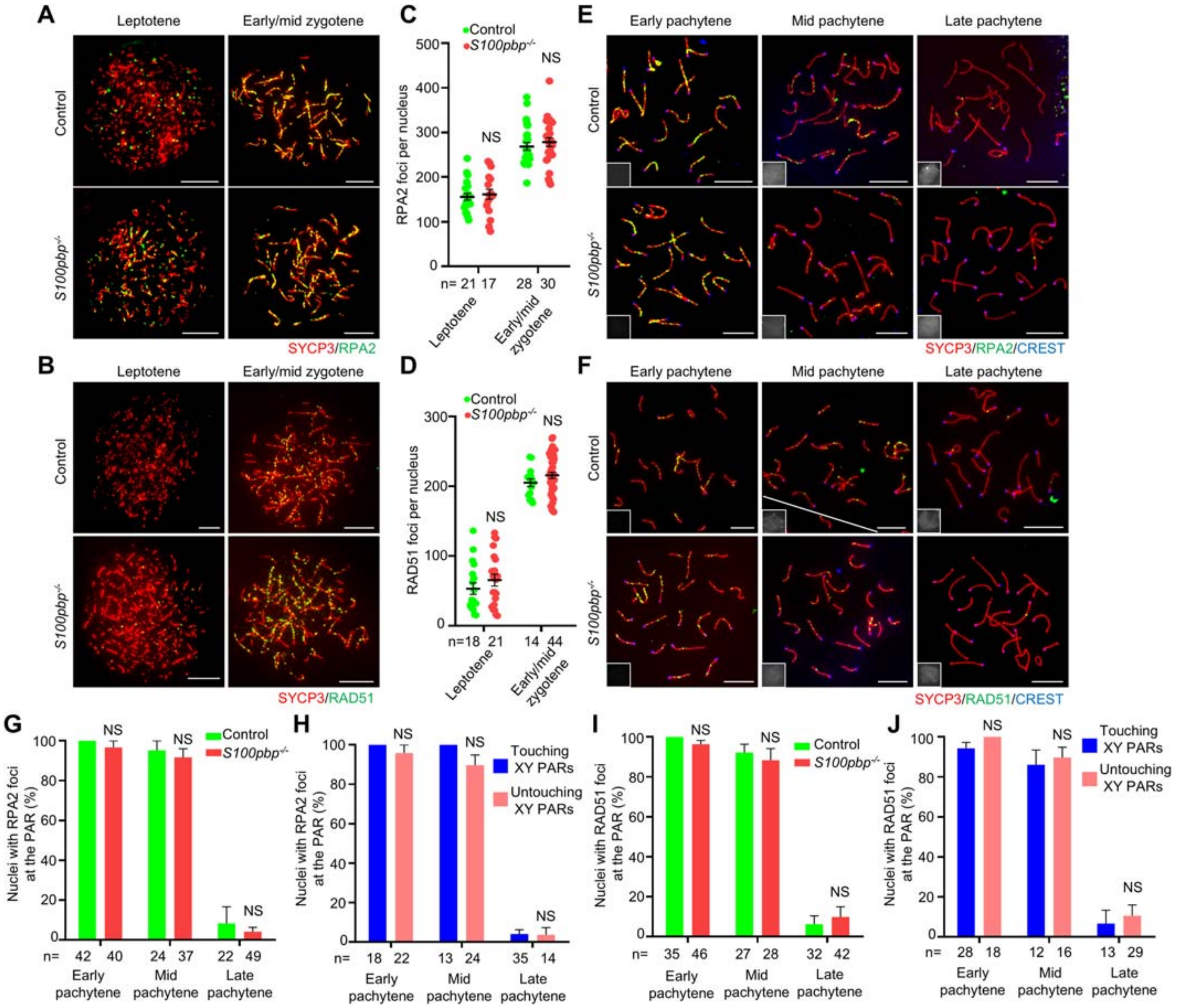

Figure EV2. The dynamics of RPA2 and RAD51 foci in *S100pbp*$^{-/-}$ spermatocytes.

(**A**, **B**) Immunofluorescence staining with antibodies against SYCP3 (red) and RPA2 (green, **A**) or RAD51 (green, **B**) on spermatocyte spreads. Scale bars, 10 μm. (**C**, **D**) Scatter plots showing the number of RPA2 foci (**C**) and RAD51 foci (**D**) per nucleus in control and *S100pbp*$^{-/-}$ spermatocytes at the indicated stages. *n*, the number of cells scored from at least three biological replicates. NS, not significant; two-tailed Student's *t* test. (**E**, **F**) Immunofluorescence staining with antibodies against SYCP3 (red), CREST (blue), and RPA2 (green, **E**) or RAD51 (green, **F**) on spermatocyte spreads. Miniaturized H1t staining (gray) images are shown in the lower-left corner of the overlay images. Scale bars, 10 μm. (**G**, **I**) Frequencies of nuclei with RPA2 foci (**G**) and RAD51 foci (**I**) were detected at the pseudoautosomal region (PAR) in spread early, mid, and late pachytene spermatocytes. (**H**, **J**) Frequencies of nuclei with RPA2 foci (**H**) and RAD51 foci (**J**) detected at the PAR were compared between nuclei with touching XY PARs and those with untouching XY PARs in *S100pbp*$^{-/-}$ mice. For (**C**), (**D**), and (**G–J**), data represent the mean ± SEM from at least three biological replicates. *n*, the number of cells scored. NS, not significant; two-tailed Student's *t* test.

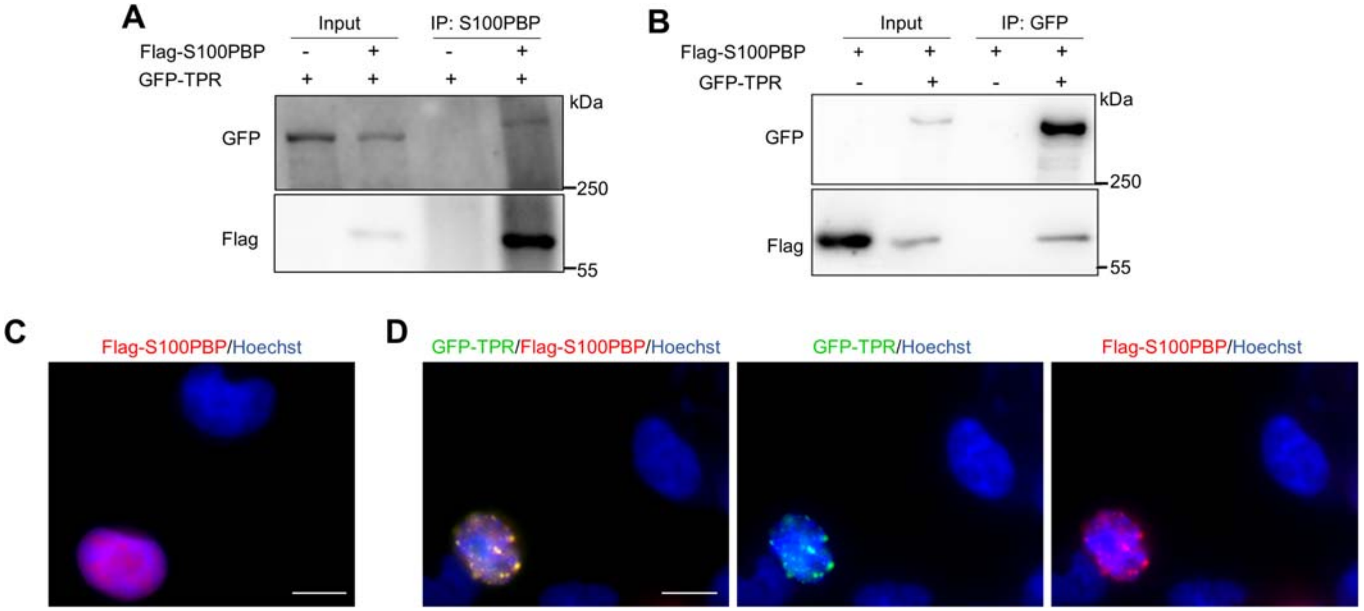

Figure EV3. Co-immunoprecipitation (Co-IP) and co-immunofluorescence staining confirmed the interaction and co-localization between S100PBP and TPR exogenously expressed in cultured HEK-293T cells.

(**A**, **B**) Co-IP was performed using an anti-S100PBP antibody (**A**) and an anti-GFP antibody (**B**) in HEK-293T cells that were exogenously expressing S100PBP (with an N-terminal Flag tag) and TPR (with an N-terminal GFP tag), followed by western blotting with the anti-Flag and anti-GFP antibodies. (**C**, **D**) Immunofluorescence staining with antibodies against Flag (red) and GFP (green) antibodies in HEK-293T cells exogenously expressing Flag-S100PBP (**C**), or Flag-S100PBP and GFP-TPR (**D**). The nuclei were counterstained with Hoechst 33342 (blue). Scale bars, 10 μm.

© The Author(s)

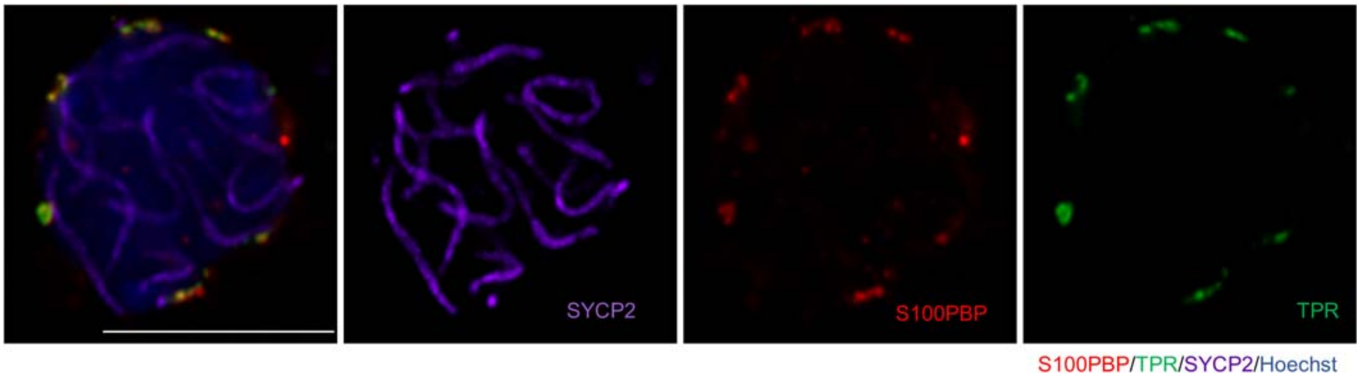

Figure EV4. S100PBP and TPR are co-localized in oocytes.

Representative confocal imaging of zygotene oocytes on the oocyte smear of fetal ovaries from wild-type mice (16.5 dpc) after immunofluorescence staining for TPR (green), S100PBP (red) and SYCP2 (purple). The nuclei were counterstained with Hoechst 33342 (blue). Scale bars, 10 µm.

© The Author(s)

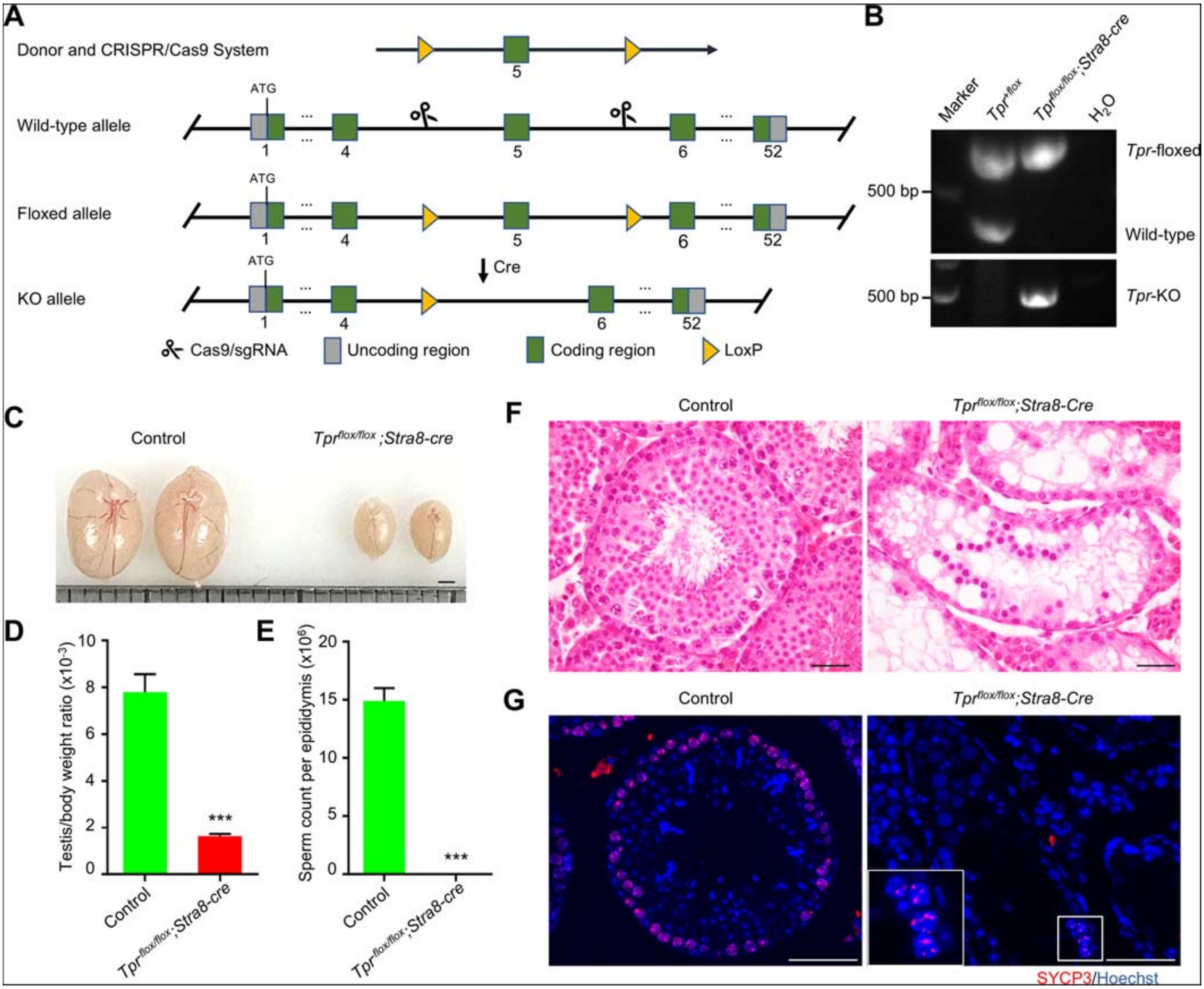

Figure EV5. Generation and spermatogenic analysis of *Tpr*$^{flox/flox}$*;Stra8-cre* mice.

(**A**) The strategy to generate *Tpr*$^{flox/flox}$*;Stra8-cre* mice. (**B**) PCR analysis of genomic DNA confirmed the *Tpr* mutation in *Tpr*$^{flox/flox}$*;Stra8-cre* testes. KO, knockout. (**C**) Representative images of testes from 8-week-old control and *Tpr*$^{flox/flox}$*;Stra8-cre* mice. Each grid represents 1 mm. (**D**, **E**) The ratio of testis/body weight (**D**) and sperm count per epididymis (**E**) of 8-week-old control and *Tpr*$^{flox/flox}$*;Stra8-cre* mice. The data are from at least three biological replicates and represent the mean ± SEM. ***$P = 0.0002$ (**D**, **E**); two-tailed Student's *t* test. (**F**) Testicular histology from 8-week-old control and *Tpr*$^{flox/flox}$*;Stra8-cre* mice. Scale bars, 50 µm. (**G**) Immunofluorescence staining of testicular sections from control and *Tpr*$^{flox/flox}$*;Stra8-cre* with antibodies against SYCP3 (red), a marker of spermatocyte. The nuclei were stained with Hoechst 33342 (blue). The magnified view of the boxed area is shown in the lower-left corner of the image. Scale bars, 50 µm.

© The Author(s)
